# Metal-based nanoparticles for reprogramming macrophage polarization: Advances in immunomodulatory nanotherapeutics

**DOI:** 10.1016/j.ijpx.2026.100564

**Published:** 2026-05-07

**Authors:** Xing Du, Kai Ding, Zehao Mao, Jiangyu Li, Yongmei Zhao, Tianqing Liu

**Affiliations:** a School of Pharmacy, Nantong University, Nantong, China; b School of Science, Western Sydney University, Penrith, NSW 2751, Australia

**Keywords:** Metal-based nanoparticles, Macrophages, Disease treatment

## Abstract

Macrophages, key players in the immune system, exhibit remarkable plasticity, polarizing into pro-inflammatory (M1) or anti-inflammatory (M2) phenotypes in response to microenvironmental cues. This functional dichotomy is pivotal in the pathogenesis and progression of a wide array of diseases, including cancer, autoimmune disorders like rheumatoid arthritis, atherosclerosis, and tissue injury. Consequently, modulating macrophage polarization has emerged as a promising therapeutic strategy. Metal-based nanoparticles (MNPs) have garnered significant attention in this field due to their unique physicochemical properties, which enable them to function as both immunomodulatory agents and sophisticated drug delivery vehicles. This review provides a comprehensive overview of recent advancements in utilizing various MNPs including those based on gold (Au), silver (Ag), iron oxide (Fe₃O₄), manganese dioxide (MnO₂), titanium dioxide (TiO₂), molybdenum disulfide (MoS₂), zinc oxide (ZnO), and calcium carbonate (CaCO₃) to reprogram macrophage polarization for therapeutic benefit. We critically examine the distinct strategies employed; for instance, promoting a shift toward the M1 phenotype using Fe₃O₄ or TiO₂ nanoparticles to enhance anti-tumor immunity, versus inducing M2 polarization with Au or Ag nanoparticles to resolve inflammation and promote tissue repair. The underlying mechanisms, such as reactive oxygen species (ROS) modulation, targeted delivery of therapeutic payloads, and direct interaction with cellular signaling pathways, are discussed in detail. Finally, this review summarizes the therapeutic potential of MNPs in macrophage-targeted immunomodulation and highlights the existing challenges and future perspectives for their clinical translation.

## Graphical abstract

**Figure:**
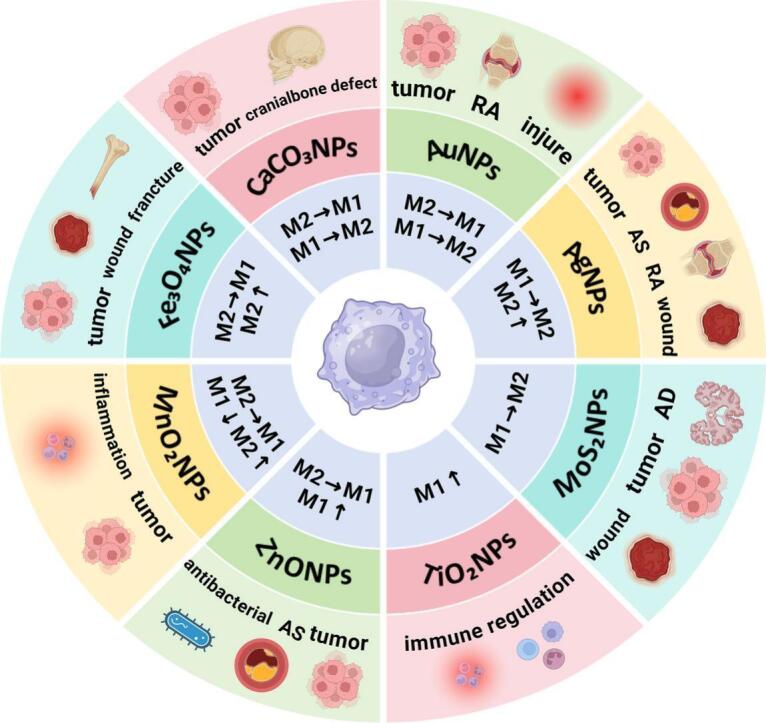

## 1 Introduction

In the immune system, macrophages play a crucial role in both innate and cellular immunity. They phagocytose pathogens and debris, and activate lymphocytes to initiate immune responses (Guilliams and Scott, 2022; Jenkins and Allen, 2021). Macrophage origins vary, with tissue-resident ones potentially arising from embryonic yolk sac progenitors, monocytes, or mixed sources, while tumor-associated macrophages (TAMs) mainly derive from circulating monocytes (Jenkins and Allen, 2021; Wang et al., 2022; Mi et al., 2022; Borst et al., 2021; Zhao et al., 2025a; Munoz et al., 2021). When stimulated, natural macrophages can polarize into two extreme phenotypes. One type is the M1 macrophage, also known as the classically activated or pro-inflammatory macrophage. M1 macrophages can be activated by toll-like receptor (TLR) ligands such as LPS, IFN-γ, TNF-α, and GM-CSF. These macrophages produce pro-inflammatory cytokines (IL-1β, IL-6, IL-12, IL-18, and IL-23), mediate resistance to pathogens, exhibit strong microbicidal and tumoricidal activities, generate high levels of reactive nitrogen species and intermediates, and promote Th1 responses (Wang et al., 2019; Xie and Ouyang, 2023; Yunna et al., 2020; Zhao et al., 2021; He et al., 2024). The other type is the M2 macrophage, also referred to as the alternatively activated or anti-inflammatory macrophage (Wang et al., 2019; Ni et al., 2021). M2 macrophages can be further divided into four subtypes. M2a macrophages, activated by IL-4 and IL-13, are characterized by the expression of the receptor CD206 on the cell surface and are involved in tissue repair and immune regulation (Wang et al., 2019; Liu et al., 2021; Viola et al., 2019; Muñoz et al., 2020)；M2b macrophages, activated by immune complexes (IC) and LPS or IL-1β, are characterized by the expression of the receptor CD86 and primarily regulate immune responses (Wang et al., 2019; Liu et al., 2021; Viola et al., 2019; Muñoz et al., 2020)；M2c macrophages, activated by glucocorticoids, IL-10, and TGF-β, are characterized by the expression of the receptor CD163 and are mainly involved in the phagocytosis of apoptotic cells (Wang et al., 2019; Viola et al., 2019; Muñoz et al., 2020)；Finally, M2d macrophages, also known as TAMs, are activated by adenosine and TLR4 or IL-6, and can produce VEGF, which promotes angiogenesis and metastasis within tumors (Wang et al., 2022; Liu et al., 2021; Muñoz et al., 2020; Xu et al., 2022).

Macrophages stimulated by different stimuli play important roles in various diseases. In patients with severe Plasmodium falciparum pulmonary edema, the expression of the M1 macrophage marker CD40 is higher than that in patients without pulmonary edema, indicating that the induction and polarization of M1 phenotype macrophages in lung tissue are increased in patients with pulmonary edema, thereby enhancing the inflammatory response (Klinkhamhom et al., 2020; Pan et al., 2022). There are also reports that the levels of leucine-rich α-2-glycoprotein 1 (LRG1) in atherosclerotic tissue and serum samples of patients with coronary artery disease (CAD) are elevated. LRG1 stimulates the pro-inflammatory M1-like polarization of macrophages by activating the extracellular signal-regulated kinase 1/2 (ERK1/2) and c-Jun N-terminal kinase (JNK) pathways, thereby promoting atherosclerosis (Wang et al., 2024a; Cheng et al., 2021a). Contrary to the conventional view, Zhou ZY and colleagues found that in hepatocellular carcinoma, M1-like TAMs produce the anti-inflammatory cytokine IL-1β, which induces the production of PD-L1, thereby exerting a tumor-promoting effect (Zong et al., 2019; Wang et al., 2024b). M2 macrophages are intricately related to pulmonary fibrosis, allergic asthma, septic complications, tumors, wound healing, attenuate secondary lymphedema, and non-alcoholic fatty liver disease (Wang et al., 2021a; Chen et al., 2024a; Zhong et al., 2023; Chen et al., 2024b; Chen et al., 2023a; Liu et al., 2024a; Ding et al., 2021; Zhou et al., 2023a). M2a macrophages play important roles in fibrosis in endometriosis and in influencing the outcomes after cerebral ischemic stroke (Chen et al., 2025; Huang et al., 2024; Shi et al., 2022). Cao WQ et al. believe that esophageal adenocarcinoma (EAC) cells can polarize THP1 cells into M2d macrophages to promote the migration and invasion of EAC cells (Cao et al., 2015). In the study by Tang LX et al., it was found that M2c macrophages mediate the elevation of IL-10 levels in mouse lung tissue by activating the JAK1/STAT3/SOCS3 signaling pathway, thereby reducing lung injury and protecting lung tissue (Tang et al., 2017). In addition, Min Zhao et al. found that Huoxue Tongfu formula can induce M2c macrophages through the MerTK/PI3K/AKT pathway to eliminate NETs in peritoneal adhesions (Zhao et al., 2024).

Definition of Nanoparticles (NPs): Nanoparticles (NPs) are minute particles composed of materials such as latex, polymers, ceramic particles, metal particles, and carbon particles, typically ranging in size from 1 to 100 nm (Cheng et al., 2021b). Due to their physicochemical properties, including chemical composition, size, shape, binding, morphology, and surface properties, NPs are increasingly being utilized in medical applications. Their surfaces can be hydrophilic or hydrophobic substances and exhibit surface charge and specific ligands, which are reference factors for selecting nanomaterials for the treatment of clinical diseases.

Advantages of metal Nanoparticles (MNPs): Metal nanoparticles enhance medical drug delivery by increasing stability and bioavailability, enabling precise targeting to disease sites (passive or active), and providing efficient drug loading due to large surface area (Ren et al., 2025). Metal nanoparticles can release drugs via stimuli or light activation, reduce toxicity by targeting diseased tissues, and combine delivery with imaging and therapy. Additionally, some metal nanoparticles possess antibacterial properties. Their biocompatibility and tunable properties further improve efficacy, reduce side effects, and enable multifunctional applications (Scafa Udriște et al., 2024).

Classification: Based on the primary components of nanoparticles, we categorize them into lipid nanoparticles, natural nanoparticles (such as extracellular vesicles (EVs)), polymeric nanoparticles, and inorganic nanoparticles. Lipid nanoparticles consist of various subtypes, but the most typical structure is spherical, including a lipid bilayer with at least one cavity in the center. These are commonly used to deliver nucleic acid genetic materials such as mRNA and siRNA and represent a frequently employed class of drug delivery nanoparticles. Polymeric nanoparticles can be synthesized from natural or synthetic materials, as well as from monomers or preformed polymers. They can encapsulate therapeutic drugs within the nanoparticle core, embed them in a polymeric matrix, or chemically conjugate or attach them to the polymer or nanoparticle surface. This enables a wide range of payloads, including hydrophobic and hydrophilic compounds, and substances with varying molecular weights, such as small molecules, biomacromolecules, proteins, and vaccines, to be combined with polymers for use. Inorganic nanoparticles are synthesized using metals like gold, silver, iron, and titanium, or inorganic materials such as silicon. Due to the inherent properties of their base materials, inorganic nanoparticles possess unique physical, electrical, magnetic, and optical characteristics. For example, iron oxide, a common metal material, is also used to make contrast agents because of its magnetism. The focus of this article is on the application of metal-based nanoparticles in diseases related to macrophage polarization (Gupta et al., 2021; Hou et al., 2021). Finally, this review summarizes the therapeutic potential of MNPs in macrophage-targeted immunomodulation and highlights the existing challenges and future perspectives for their clinical translation. To ensure the transparency and reproducibility of our literature synthesis, the detailed bibliographic search strategy is provided in Supporting Information (Table S1 and S2). Furthermore, acknowledging the diversity in macrophage nomenclature, we have provided a technical breakdown of our phenotypic categorization in Supporting Information. This includes Table S3, which maps specific marker panels (e.g., CD86, CD206) and functional readouts to their interpreted phenotypes while highlighting critical pitfalls such as marker overlap and the context-dependent nature of macrophage plasticity in diseases like cancer and rheumatoid arthritis.

## 2 Strategies for conventional disease treatment using macrophage polarization

### 2.1 Mechanistic regulation of tumor-associated macrophage (TAM) Plasticity

TAMs are pivotal architects of the tumor microenvironment (TME), often constituting up to 50% of the total tumor mass (Larionova et al., 2020). The high degree of TAM plasticity makes them a primary target for immunotherapy, as their polarization state significantly dictates clinical outcomes (Tang et al., 2021). While M1-polarized macrophages exert potent anti-tumor effects through the secretion of tumor necrosis factor-alpha (TNF-α) and reactive oxygen species (ROS), the TME predominantly fosters the M2 phenotype, which promotes tumor growth and immune evasion via interleukin-10 (IL-10) and transforming growth factor-beta (TGF-β) (Che et al., 2024; Qian et al., 2023; Zhao et al., 2025b; Wang et al., 2025a). Beyond these biochemical signals, the physical properties of the TME—including tumor stiffness, density, and hypoxia—critically impede immune cell infiltration and therapeutic efficacy (Dong et al., 2026). Consequently, therapeutic strategies have evolved from simple macrophage depletion toward the precise molecular reprogramming of M2-to-M1 phenotypes.

A critical axis in this regulatory network is the colony-stimulating factor-1 (CSF-1) and its receptor (CSF-1R), which govern macrophage survival, migration, and differentiation (Kapanadze et al., 2023; Tomassetti et al., 2024). In many malignancies, the overexpression of CSF-1 correlates directly with increased TAM infiltration and poor prognosis (Wyrobnik et al., 2023; Cao et al., 2025; Qin et al., 2025). However, critical evaluation of current CSF-1/CSF-1R inhibitors reveals a therapeutic gap; for instance, while agents like Emactuzumab (alone or with paclitaxel) effectively eliminate M2-like macrophages, they fail to actively reprogram them into the anti-tumor M1 phenotype (Kapanadze et al., 2023). In contrast, CSF-1R inhibitors like LY3022855 aim to reverse pro-tumor macrophage accumulation by directly blocking the fundamental monocyte/macrophage functions necessary for tumor support (Tomassetti et al., 2024). The upstream regulation of this pathway further complicates the TME; in hepatocellular carcinoma (HCC), the overexpression of lncRNA HOMER3-AS1 drives CSF-1 secretion to induce M2 polarization, while MMP-21 acts as a multifaceted recruiter by upregulating CCL-14, CSF-1, and FGF-1 to sustain the M2-dominant landscape (Autio et al., 2020; Pu et al., 2021; He et al., 2021; Zhou et al., 2023b).

**Fig. 1:**
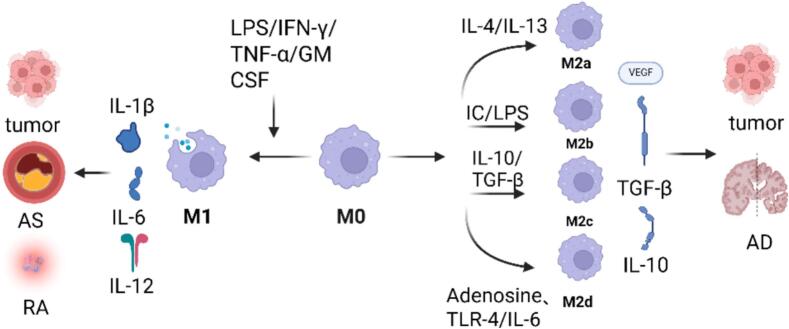
Polarization pathways of macrophages and their roles in diseases.

Beyond receptor-ligand signaling, the metabolic landscape of the TME specifically the accumulation of lactate serves as a secondary, yet equally potent, driver of M2 polarization. Emerging evidence suggests that lactate functions as a signaling molecule through the lactate-MCT-HIF1α and lactate-ERK-STAT3 pathways to reinforce the immunosuppressive state (Ajam-Hosseini et al., 2024; Zhang and Li, 2020; Mu et al., 2018). This metabolic-immunologic link provides a dual-targeted therapeutic opportunity: inhibiting lactate production not only slows tumor glycolysis but also disrupts the signals that promote M2 differentiation. This is exemplified by the role of the lactate sensor Gpr132; its absence has been shown to reduce M2 macrophage populations, thereby preventing metastasis and inhibiting the glycolytic flux that fuels tumor progression (Zhang et al., 2021; Chen et al., 2017; Tao et al., 2022). Ultimately, these findings suggest that an effective “repolarization” strategy must look beyond single receptors and instead address the synergistic crosstalk between genetic regulators like lncRNAs, enzymatic recruiters like MMP-21, and metabolic drivers like lactate (See Fig. 1).

### 2.2 Macrophage heterogeneity and therapeutic targets in rheumatoid arthritis

The immune system maintains physiological homeostasis through inflammation, a sophisticated response to injury, infection, and allergens. However, dysregulation in this system, often stemming from genetic defects in inflammatory components, can lead to chronic susceptibility to disease. Macrophages are central to this process, acting as primary orchestrators across the initiation, progression, resolution, and repair phases of inflammation. This is particularly evident in Rheumatoid Arthritis (RA), a systemic inflammatory disorder characterized primarily by joint destruction, yet also associated with severe multi-organ complications in the heart, lungs, eyes, and brain. Notably, cardiovascular manifestations—such as accelerated atherosclerosis and heart failure—remain the leading cause of mortality in RA patients (Sanghavi et al., 2024).

At the cellular level, the RA synovium is characterized by a dense infiltration of synovial macrophages (Kai et al., 2024). These cells exist in a dynamic equilibrium between two functional states: the pro-inflammatory M1 phenotype, which drives joint erosion through the secretion of cytokines such as TNF-α, IL-1β, and IL-6, and the anti-inflammatory M2 phenotype, which promotes tissue repair via IL-10 and TGF-β. In active RA, this balance is significantly skewed toward the M1 phenotype (Kai et al., 2024; Cutolo et al., 2022). Recent research has focused on identifying the specific molecular drivers of this M1 dominance. For instance, the long non-coding RNA lncRNA H19 is significantly upregulated in RA patients and murine models, where it appears to work in tandem with the histone demethylase KDM6A to lock macrophages into a pro-inflammatory state. This pathway provides a precise target for intervention, as the KDM6A inhibitor GSK-J4 has been shown to downregulate lncRNA H19 and mitigate arthritis progression (Zhu et al., 2021).

Complementary to epigenetic regulation, targeting classical signaling and inflammasome pathways offers further therapeutic avenues to suppress M1-mediated damage. While the NF-κB pathway remains a central driver of macrophage activation, recent findings by Gaoran Ge’s team demonstrate that punicalagin can specifically block this pathway, thereby inhibiting both M1 polarization and the catastrophic process of pyroptosis (Ge et al., 2022). Furthermore, the precision of RA therapy can be enhanced by targeting the NLRP3 inflammasome, a key executor of the inflammatory response. Licochalcone B has emerged as a promising candidate in this regard, as it selectively inhibits NLRP3 activation by disrupting the critical NEK7-NLRP3 interaction (Li et al., 2022). Collectively, these studies suggest that a multifaceted approach—addressing epigenetic drivers (lncRNA H19), classical signaling (NF-κB), and inflammasome assembly (NLRP3)—is essential for restoring macrophage homeostasis and resolving the systemic burden of RA.

### 2.3 Macrophage heterogeneity and therapeutic programming in atherosclerosis

Atherosclerosis (AS) is a multifaceted disease of the medium and large arteries, fundamentally driven by lipid deposition and sustained chronic inflammation (Ma et al., 2021). As a primary etiology of global cardiovascular disease (CVD), AS underpins life-threatening conditions such as ischemic heart disease, stroke, and peripheral vascular disease (Tabares-Guevara et al., 2021). The hallmark of its progression is the formation of the atherosclerotic plaque—a complex architectural entity consisting of a necrotic lipid core, calcified deposits, and a diverse cellular infiltrate including smooth muscle cells, damaged endothelial cells, and immune cells. While current clinical management relies heavily on lifestyle modifications and pharmacological control of risk factors—such as statins, antiplatelet agents, and ACE inhibitors—emerging research suggests that modulating the local immune microenvironment may offer more precise therapeutic outcomes.

The microenvironment of AS lesions is characterized by a dense aggregation of activated immune cells and the accumulation of oxidatively modified lipoproteins. Within this milieu, the phenotypic balance of macrophages serves as a critical determinant of plaque vulnerability. In unstable, rupture-prone plaques, M1 pro-inflammatory macrophages predominate, particularly in the adventitia and the thin fibrous caps (Momtazi-Borojeni et al., 2019; Yu et al., 2023). These M1 cells accelerate endothelial dysfunction and necrotic core expansion by generating reactive oxygen species (ROS) and pro-inflammatory cytokines such as TNF-α and IL-6 (Eshghjoo et al., 2022). Conversely, stable plaques are often dominated by M2 anti-inflammatory macrophages, which exhibit reduced foam cell formation and contribute to plaque stability through the production of large granular calcified deposits (Peng et al., 2020). Consequently, the M1/M2 ratio has emerged as a predictive indicator of plaque progression and clinical prognosis.

Targeting this M1/M2 rheostat has become a focal point for novel pharmacological interventions, with several natural products and traditional formulations demonstrating potent regulatory capabilities. For instance, curcumin achieves M1-to-M2 transformation by specifically inhibiting the TLR4/NF-κB pathway and suppressing MAPK phosphorylation (Momtazi-Borojeni et al., 2019). Similarly, the traditional formula Huanglian Jiedu Decoction (HLJDD) has been shown to stabilize plaques by downregulating M1 markers like iNOS and CD86 while simultaneously promoting M2 markers such as Arg-1 and CD163 (Cai et al., 2021). Building on these findings, more complex interventions like Busengkangshuai tablets utilize a multi-pathway approach—activating autophagy proteins and inhibiting the PI3K/AKT/mTOR axis—to suppress the M1 phenotype through PPAR-γ/NF-κB modulation (Xie et al., 2020). The precision of these therapies is further exemplified by Ganoderma lucidum triterpenoids, which provide a targeted blockade of the TLR4/MyD88/NF-κB signaling cascade (Quan et al., 2024). Furthermore, the discovery that dihydromyricetin (DMY) regulates the miR-9/SIRT1/NF-κB axis suggests that microRNA-mediated epigenetic control is a viable target for shifting the macrophage balance toward an atheroprotective M2 state (Yang et al., 2023).

## 3 Treatment of diseases using metal nanoparticles to modulate macrophage polarization

In this section, we discuss the diverse roles of MNPs in regulating macrophage fate. It is important to note that the strength of mechanistic evidence supporting these pathways varies across the literature. To ensure a rigorous synthesis, we have implemented an Evidence-Grading Scheme (EGS) to differentiate between causal validation and correlative observations (detailed in Table S4, Supporting Information). This framework is applied throughout the following discussions to clarify the degree of certainty for each proposed mechanism.

### 3.1 Advanced functionalization of gold nanoparticles: mechanisms and applications in macrophage-mediated tissue repair and oncology

**Fig. 2:**
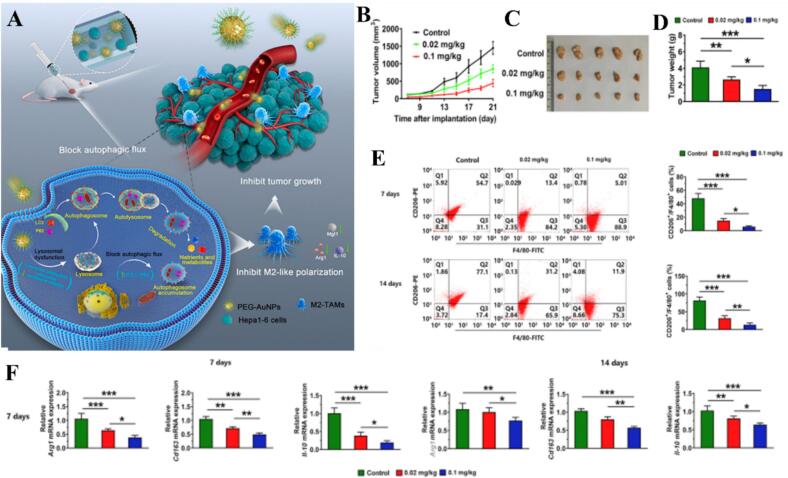
In vivo antitumor activity and inhibition of M2 macrophage polarization by PEG-AuNPs. (A) PEG-AuNPs suppressed TAMs M2 polarization, elicited antitumor immunotherapy and inhibited subcutaneous tumor growth in mice. (B) Tumor growth curves. Hepa1–6 cells were subcutaneously inoculated into the right flank of male BALB/c mice with or without 20 nm PEG-AuNPs, and the tumor growth was monitored for 21 days. (C) The gross images of excised tumors from the mice at the endpoint. (D) Tumor weight at the endpoint. (E) CD206+/F4/80+ macrophages were analyzed using flow cytometry in tumor tissues on Days seven and 14 after implantation. (F) The mRNA levels of genes (M2 phenotype) measured using RT-qPCR in tumors on Days seven and 14 after implantation in mice. Reference (Zhang et al., 2022) with permission from Copyright © 2025, Advanced Science.

Gold nanoparticles (AuNPs) have emerged as premier tools in immunomodulation due to their high surface-area-to-volume ratio, ease of surface functionalization, and superior tissue penetration (Niżnik et al., 2024; Yang et al., 2021a). Beyond their traditional roles as antimicrobial agents or biosensors, AuNPs serve as precise regulators of macrophage polarization rather than just passive carriers. It is essential to distinguish the intrinsic material driven effects of the gold core from those mediated by surface ligands or drugs. The gold core provides functional advantages that simple delivery cannot achieve. For example, particle geometry exerts mechanical cues on the cell membrane, where shapes like nanostars trigger distinct mechanotransduction pathways. Additionally, localized surface plasmon resonance allows for remote controlled modulation. Using near infrared light to generate local heat can facilitate M2 to M1 shifts by activating heat shock proteins. Finally, the gold surface acts as a multivalent platform for dense ligand grafting. This promotes the cross link of surface receptors with higher avidity than free molecules, transforming a passive carrier into an active signaling stimulus. Beyond surface functionalization, the gold core serves as a stable platform for multivalent interactions with membrane receptors. For instance, curcumin-functionalized AuNPs have been shown to effectively suppress M1 polarization by interfering with the TLR4-mediated recruitment of MyD88, subsequently inhibiting the phosphorylation and nuclear translocation of the NF-κB p65 subunit. This blockade results in a significant downregulation of pro-inflammatory mediators such as TNF-α and iNOS, facilitating a transition toward a reparative M2 phenotype (Zhou et al., 2023b). To clarify these distinctions, Table S7 in the Supporting Information provides a systematic analysis of core driven versus ligand driven polarization across representative studies. Current research increasingly focuses on utilizing these physical and mechanical properties to dictate cellular behavior (Chowdhury et al., 2022). For instance, Heemin Kang's team pioneered a heterodimeric nanostructure that achieves M1/M2 balance through reversible RGD uncaging/caging, demonstrating that mechanical adhesion cues are as vital as chemical signals (Kang et al., 2018). This mechanical influence is further supported by Shouning Yang's work with AuNPs and Au@CaCO_3_NPs, where macrophage elongation induced by the particles themselves served as a physical trigger for M2 polarization, whereas rounded morphologies driven by the carbonate shell favored an M1 pro-inflammatory state (Yang et al., 2021b). In the context of chronic and acute inflammatory diseases, AuNPs are increasingly designed to sequester inflammatory mediators or interfere with metabolic and epigenetic pathways. In acute lung injury (ALI), targeting the lung microenvironment via hexapeptide-coated P12-AuNPs has shown efficacy in reducing M1 populations, a mechanism further refined by ICAM-1-AFM41a-AuNPs, which utilize targeted delivery to block p65 citrullination and drive the M1-to-M2 transition (Wang et al., 2020; Yu et al., 2025). Similarly, P12-functionalized AuNPs have been adapted for sepsis-associated encephalopathy (SAE), where they form a “protein corona” to sequester systemic inflammatory proteins (Song et al., 2025a). The therapeutic scope extends to diabetic complications, where miR-155 antagonist-coupled AuNPs reduce cardiac inflammation, and periodontitis, where green-synthesized AuNPs or Nell-1/AuNP composites stabilize the oral inflammatory microenvironment to promote bone repair (Jia et al., 2017; Dannewitz et al., 2021; Ni et al., 2019a; Liao et al., 2024; Gao et al., 2022). These studies underscore that the success of AuNPs in tissue repair—whether in the gums, bone, or skeletal muscle—depends on their ability to transition macrophages from an inflammatory phase to a reparative M2 state (Bai et al., 2021; Raimondo and Mooney, 2018; Xu et al., 2025a). Notably, thioctic acid-modified LA-Au NPs illustrate a sophisticated shift toward metabolic reprogramming, utilizing enhanced mitochondrial oxidative phosphorylation to drive muscle regeneration (Xu et al., 2025a; Xu et al., 2025b). Similar reparative transitions are observed in rheumatoid arthritis through Triam-AuNPs and in infectious wound healing where PHMB@Au NPs and TPA-Py@AuNCs (TAB NPs) combine antibacterial action with M2 induction (Park et al., 2020; Xu et al., 2025c; He et al., 2022). Furthermore, engineered M-Fe_3_O_4_/Au nanoparticles have demonstrated the ability to combat bacterial infections while promoting bone regeneration through the synchronized production of ROS and induction of M2 polarization (Fu et al., 2021). Conversely, in the field of oncology, the objective shifts toward reversing the immunosuppressive tumor microenvironment (TME). Critical analysis of recent literature suggests a transition from simple drug delivery to “smart” responsive platforms. While PEG-AuNPs focus on inhibiting M2 polarization via autophagy intervention, newer systems like AuNPs targeting m6A methylation levels can block the ATG5 pathway to suppress prostate cancer growth (Zhang et al., 2022; Hao et al., 2025). For the in vivo antitumor activity and M2 macrophage polarization inhibition by PEG-AuNPs, refer to Fig. 2. Au@PG nanoparticles have been shown to induce M1 polarization in lung cancer treatment, effectively transforming “cold” tumors into immunogenic “hot” microenvironments (Su et al., 2022). The integration of external stimuli—such as radiation, light, or ultrasound—represents the current frontier. Au@SiO_2_ and CpG@Au NPs act as radiosensitizers to amplify M1 polarization, while photothermal and sonodynamic catalysts like Au/PDA/HRP@DLP and HCP nanocomposites trigger systemic antitumor immunity. Advanced hybrids such as PSP-AuNPs, MTX/Au@PVCL NGs, UGsGP, and Au/CuNDs/DOX@NS leverage dual-mode imaging and responsive ROS production to repolarize TAMs (Leng et al., 2024; Chen et al., 2023b; Jia et al., 2023; Cao et al., 2021; Song et al., 2025b; Zhang et al., 2025a; Feng et al., 2025; Wang et al., 2025b). Furthermore, glycoglycan-based platforms, such as galactosylated Au@PGlyco NPs and Glyco-GNPs, demonstrate the versatility of AuNPs; these particles can promote antitumorigenic M1 activity in cancers while shifting toward restorative phenotypes in autoimmune contexts (Chia et al., 2021; Fernandez Alarcon et al., 2025; Cheng et al., 2025). This adaptability is echoed in wound care, where BSA-(Au@Ag/CeO_2) and Ti3C2/Au NPs synchronize antibacterial efficacy with tissue-reparative M2 polarization (Chen et al., 2023c; Guo et al., 2025). Finally, the engineering of the “nanocorona” via IgG-adsorbed Au Nanostars (AuS) represents a sophisticated move toward remodeling protein structures to ensure durable immune memory, proving that AuNPs are no longer passive carriers but active orchestrators of the immune response (Park et al., 2025). In conclusion, gold nanoparticles have evolved from passive drug carriers into sophisticated immunomodulatory agents capable of precise macrophage reprogramming. By integrating mechanical cues, surface ligand engineering, and external stimuli-responsive designs, AuNPs can effectively steer macrophage fate—promoting reparative M2 phenotypes for tissue regeneration and chronic inflammation resolution, or driving pro-immunogenic M1 phenotypes to overcome tumor immunosuppression. This functional versatility, underpinned by emerging insights into metabolic and epigenetic regulation, underscores the immense potential of AuNPs as a cornerstone for next-generation precision immunotherapy (Table 1, Table 1).

**Table 1:** AuNPs act on macrophages to treat various diseases.

Nanoparticle	Effect on Macrophages	Key Mechanism	Disease Application	References
RGD-AuNPs-MNCs	Promotes M2, inhibits M1	Magnetic-controlled RGD release enhances macrophage adhesion	Inflammation modulation, tissue repair	(Kang et al., 2018)
Au@CaCO3NPs	Induces M1 polarization	Alters macrophage morphology and promotes M1 marker secretion	Immunomodulation, anti-tumor	(Yang et al., 2021b)
PEG-AuNPs	Suppresses M2, promotes M1	Autophagy intervention inhibits TAMs M2 polarization	Cancer immunotherapy	(Zhang et al., 2022)
miR155-AuNPs	Promotes M2 polarization	miR155 delivery reduces inflammatory responses	Diabetic cardiomyopathy	(Jia et al., 2017)
P12 peptide-AuNPs	Promotes M2, reduces M1	TLR inhibition modulates inflammatory cytokines	Acute lung injury (ALI)	(Wang et al., 2020)
AuNPs (bone repair)	Promotes M2 polarization	Increases M2 macrophage population to enhance osteogenesis	Bone injury repair	(Bai et al., 2021)
IL-4-AuNPs (PA4)	Promotes M2a polarization	IL-4 conjugation enhances macrophage phenotype switching	Skeletal muscle injury repair	(Raimondo and Mooney, 2018)
PSP-AuNPs	Modulates immune function	Reduces TNF-α, elevates IL-10 to enhance anti-tumor immunity	Liver cancer therapy	(Leng et al., 2024)
Au@SiO2NPs + XR	Promotes M1 polarization	ROS amplification and caspase-3 activation promote macrophage-mediated tumor clearance	Glioblastoma	(Chen et al., 2023b)
Triam-AuNPs	M1 → M2 repolarization	Modulates inflammatory factors in synoviocytes and macrophages	Rheumatoid arthritis	(Park et al., 2020)
AuNPs (periodontitis)	Induces M2 polarization to balance M1/M2	Regulates inflammatory factors and improves periodontal microenvironment	Periodontitis	(Gao et al., 2022)

**Table 1:** (continued).

Nanoparticle	Effect on Macrophages	Key Mechanism	Disease Application	References
Au@PG NPs	Promotes M1 polarization	ER stress→SYK activation enhances immunomodulation	Lung cancer immunotherapy	(Su et al., 2022)
Fe₃O₄/Au NPs + MW	Promotes M2 polarization	Microwave-induced ·O₂^−^ generation reduces inflammatory factors	Osteomyelitis/bone regeneration	(Fu et al., 2021)
Au@PGlyco NPs	M2 → M1 conversion	ROS-mediated macrophage phenotype reprogramming	Cancer immunotherapy	(Chia et al., 2021)
Ti₃C₂/Au NPs	Promotes M2 polarization	Photothermal antibacterial effects + pro-angiogenesis	Infected wound healing	(Chen et al., 2023c)
LA-Au NPs	Promotes M2 polarization	Metabolic reprogramming (enhanced lysosomal autophagy & mitochondrial OXPHOS)	Muscle regeneration/sarcopenia	(Xu et al., 2025b)
MTX/Au@PVCL NGs	M2 → M1 repolarization	pH/H₂O₂-responsive release of MTX/AuNPs synergistically induces apoptosis	Melanoma (chemo-immunotherapy)	(Jia et al., 2023)
CpG@Au NPs + radiotherapy	M2 → M1 conversion	TLR9 activation + radiosensitization enhances T-cell response	Glioma (radio-immunotherapy)	(Cao et al., 2021)
TPA-Py@AuNCs⊂BSA (TAB NPs)	Promotes M2 polarization	Photothermal-controlled release of AIE photosensitizer (antibacterial + immunomodulatory)	Infected wound healing	(Xu et al., 2025c)
PHMB@Au NPs	M1 → M2 polarization	Photothermal antibacterial effects + pro-angiogenesis	Bacterial wound infections	(He et al., 2022)
ICAM-1-AFM41a-AuNPs	M1 → M2 polarization	ICAM-1 targeting delivers PAD2 inhibitor to block p65 citrullination	*Pseudomonas aeruginosa*-induced ALI	(Yu et al., 2025)
Glyco-GNPs (Man/Sia)	Reprograms to M1 or M2	Utilizes liver tropism; promotes M1 in cancer but restorative M2 in autoimmune disease	Liver cancer / Autoimmune biliary disease	(Fernandez Alarcon et al., 2025)
P12-AuNPs	Regulates activation	Targets peritoneal macrophages and forms a protein corona to sequester inflammatory proteins	Sepsis-associated encephalopathy (SAE)	(Song et al., 2025a)
LA-Au NPs	Promotes M2 polarization	Metabolic reprogramming via enhanced lysosomal autophagy and mitochondrial OXPHOS	Muscle regeneration / Sarcopenia	(Xu et al., 2025a)
AuNPs	Inhibits M2 polarization	Decreases m6A methylation levels to inhibit the ATG5/autophagy pathway	Prostate cancer (HSPC and CRPC)	(Hao et al., 2025)
Au/PDA/HRP@DLP	Shift to reparative phenotype	Combined PTT/SDT kills bacteria and triggers an in situ vaccine effect	Chronic osteomyelitis	(Song et al., 2025b)
IgG-adsorbed AuS	M2 → M1 remodeling	Nanocorona-engineered protein structures combined with NIR-induced PTT	Cancer vaccination / Immunotherapy	(Park et al., 2025)
Au/2ATP@PGlyco NP	M2 → M1 reprogramming	Light-driven charge transfer generates ROS to trigger NF-κB and STAT-1 signaling	Bladder cancer	(Cheng et al., 2025)
UGsGP Nanocomposites	Promotes M1 polarization	Starvation, PTT, and gas therapy cascade redefines the tumor microenvironment	Primary and distant tumor therapy	(Zhang et al., 2025a)
BSA-(Au@Ag/CeO2)	Induces M2 polarization	Synchronized ROS-mediated antibacterial action and immunomodulation	Refractory MRSA wound infections	(Guo et al., 2025)
HCP Nanocomposites	Triggers TAM polarization	Multi-enzyme nanozymes remodel TME and enhance PTT/PDT effects	Systemic antitumor immunity	(Feng et al., 2025)
Au/CuNDs/DOX@NS	Reverses immunosuppression	Photothermal-enhanced CDT promotes ROS production to drive polarization	Triple-negative breast cancer (TNBC)	(Wang et al., 2025b)

### 3.2 Advanced applications of Silver Nanoparticles: From Antimicrobial Agents to Precision Immunomodulators

**Fig. 3:**
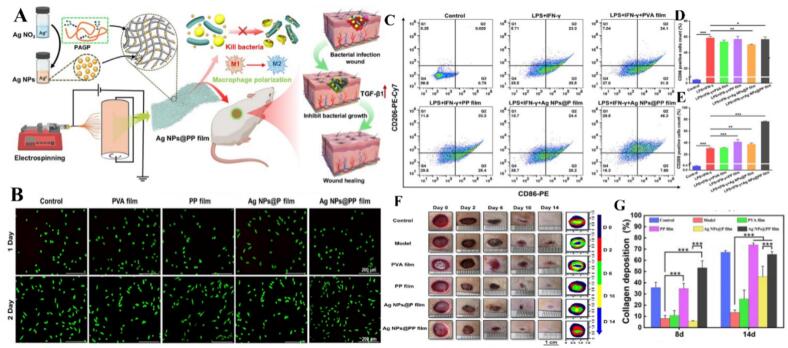
The Ag NPs@PP film demonstrated a stronger ability to promote M2 polarization of macrophages and exhibited higher efficacy in healing wounds infected by fecal bacteria. (A)Facile fabrication of Ag NPs@PP film embedding green-synthesized Ag NPs via electrospinning technology, with the enhanced wound healing for infected wound. (B) Evaluation of cell growth by live-dead cell staining after incubation of nanofiber films with L929 cells for 1 and 2 days. (C-E) Representative flow cytometry plots of RAW 264.7 phenotypes and statistics of CD86 and CD206 positive cells. (F) Representative photographs (20 ×) of infected wounds on days 0, 2, 6, 10, and 14 and schematic diagram of wound area sizes during the wound healing process. (G) Quantification of collagen coverage area. Reference (Li et al., 2025a) with permission from Copyright © 2025, Advanced Science. (For interpretation of the references to colour in this figure legend, the reader is referred to the web version of this article.)

Silver nanoparticles (AgNPs) have evolved from their traditional role as broad-spectrum antibiotics into sophisticated agents capable of orchestrating complex biological pathways for tissue repair. Historically valued for their unique optical properties and catalytic effects, their therapeutic utility was initially limited to antimicrobial activity mediated by the generation of reactive oxygen species (ROS) to induce microbial apoptosis (Raja et al., 2017; Xie, 2024; Chandraker and Kumar, 2024). However, recent critical shifts in nanomedicine emphasize that AgNPs are not merely passive toxins but active modulators of the immune microenvironment. While high concentrations can exhibit cytotoxicity in macrophages, sophisticated engineering at biocompatible doses enables precise regulation of macrophage polarization. In chronic inflammatory environments like rheumatoid arthritis (RA), where M1 macrophages drive joint destruction, folic acid-modified AgNPs (FA-AgNPs) and HA-M@PB@Ag@PD NPs specifically target these inflammatory subsets to scavenge ROS and promote a transition to the reparative M2 phenotype (Chandraker and Kumar, 2024; Hashimoto et al., 2014; Yang et al., 2021c; Su et al., 2024). This immunomodulatory capability is increasingly applied to structural regeneration; for instance, the local delivery of AgNPs enhances motor recovery after spinal cord injury by upregulating apoptotic genes in M1 macrophages, while Ag-TiO₂ nanotubes improve bone healing by inhibiting the PI3K/Akt pathway to drive M2-to-M1 polarization within tumor microenvironments (Chen et al., 2020a; Lin et al., 2023). The versatility of AgNPs is further showcased in their integration into multifunctional scaffolds designed for complex wound environments. In infected wounds, systems like Ag NCH hydrogels,Ag NPs@PP membranes, and Ag/Tlc/Csn NCs harmonize antibacterial action with the upregulation of repair factors such as bFGF and IL-10 (Zhang et al., 2023a; Li et al., 2025a; Daghian et al., 2021). The enhanced M2 polarization and wound healing efficacy of Ag NPs@PP film against fecal bacterial infection is illustrated in Fig. 3. This dual-action approach is particularly critical for chronic conditions like diabetic wounds, where spatiotemporal release systems—such as M@M-Ag-Sil-MA and the DAg/HTMS-MNs bilayer microneedle system—reprogram macrophages to resolve chronic inflammation (Mei et al., 2022; Yang et al., 2026). Similarly, Ag@GA/Gel and FA-Ag NPs/CPH utilize polyphenol-metal networks and green-synthesized particles to facilitate the M1-to-M2 transition, accelerating collagen deposition even in the presence of MRSA (Han et al., 2025; Chang et al., 2025). Beyond surface wounds, AgNPs provide innovative solutions for internal tissue engineering; for example, FS NPs loaded onto decellularized heart valves (DHVs) target M1 macrophages to reduce intracellular H2O2 levels, preventing calcification and improving valve longevity (Song et al., 2025c). Recent advancements also highlight the efficacy of composite materials like the CMC/KCG/AgNPs/APT biocryogel, which significantly increases CD163+ M2 cells to treat infected defects, and pH-responsive CMCS/PUE/Ag@CR NPs hydrogels that alleviate oxidative stress in burn healing (Li et al., 2025b; Li et al., 2025c). Even in atherosclerosis and cancer, specialized bioconjugates like CpG-AgNPs and SNP-ApAGP leverage antioxidant properties and autophagy induction to steer macrophage behavior (Raja et al., 2017; Tang et al., 2023). Collectively, these studies illustrate that by balancing their inherent antimicrobial toxicity with targeted immunomodulatory design, AgNPs have transitioned into sophisticated tools for promoting tissue regeneration and resolving systemic inflammation (Ma et al., 2020; Ren et al., 2023; Feng et al., 2016). In conclusion, silver nanoparticles have evolved from traditional broad-spectrum antibiotics into refined immunotherapeutic agents. By neutralizing oxidative stress and selectively inducing M1-to-M2 phenotypic shifts within specialized scaffolds, AgNP-based platforms provide a synergistic approach that simultaneously eradicates pathogens and orchestrates the biological pathways necessary for complex tissue repair (Table 2).

**Table 2:** AgNPs act on macrophages to treat various diseases.

Nanoparticle	Effect on Macrophages	Key Mechanism	Disease Application	References
FA-AgNPs	M1 → M2 polarization	Ag^+^ release and ROS scavenging	Rheumatoid arthritis	(Hashimoto et al., 2014)
Ag-TiO₂ nanotubes	Promotes M2 polarization	Inhibits PI3K/Akt pathway, activates autophagy	Bone repair	(Chen et al., 2020a)
AgNPs	Reduces M1 macrophages	Upregulates M1 apoptosis genes, activates PI3k-Akt pathway	Spinal cord injury	(Lin et al., 2023)
Ag NCH hydrogel	M1 → M2 polarization	Downregulates IL-6/TNF-α, upregulates IL-10	Infected wounds	(Zhang et al., 2023a)
SNP-ApAGP	M0 → M1 polarization, M2 → M1 repolarization	Enhances macrophage autophagy	Cancer treatment	(Raja et al., 2017)
CpG-AgNPs	Promotes macrophage repolarization	Enhances fatty acid oxidation metabolism	Atherosclerosis	(Tang et al., 2023)
HA-M@PB@Ag@PD NPs	M1 → M2 repolarization	ROS scavenging, induces RA-FLS apoptosis	Rheumatoid arthritis	(Su et al., 2024)
Ag NPs@PP membrane	Promotes M2 polarization	Antibacterial and immunomodulatory synergy	Bacterial-infected wounds	(Li et al., 2025a)
Ag/Tlc/Csn NCs	Promotes M2 polarization	Induces expression of repair factors (bFGF/CD206/IL-10)	Infected wounds	(Daghian et al., 2021)
M@M-Ag-Sil-MA	M1 → M2 transformation	Spatiotemporal release of Ag^+^ and metformin, inhibits NETs	Diabetic wounds	(Mei et al., 2022)
DAg/HTMS-MNs	M1 → M2 reprogramming	Heparin-mediated MCP-1 sequestration and sustained release of taurine	Diabetic wound therapy	(Yang et al., 2026)
Ag\@GA/Gel	M2 polarization	Polyphenol-metal network scavenges ROS and reduces IL-6/pro-inflammatory cytokines	Diabetic healing / Angiogenesis	(Han et al., 2025)
FA-Ag NPs/CPH	M1 → M2 transition	Eradication of MRSA and inhibition of inflammatory pathways	MRSA-infected diabetic wounds	(Chang et al., 2025)
FS NPs (on DHVs)	M1 → M2 polarization	Specific targeting of M1 macrophages to reduce intracellular H2O2 levels	Tissue-engineered heart valves	(Song et al., 2025c)
AgNPs/APT Biocryogel	M1 → M2 polarization	Significant increase in CD163+ cells (2.6×) and inhibition of TNF-α/iNOS	Infected tissue defects	(Li et al., 2025b)
CMCS/PUE/Ag\@CR NPs	M1 → M2 polarization	pH-responsive system alleviates oxidative stress via Puerarin and Ag\@CR NPs	Infected burn wounds	(Li et al., 2025c)

### 3.3 Molybdenum Disulfide (MoS₂) Nanostructures: Transitioning from Industrial Catalysts to Dynamic Immunomodulators

**Fig. 4:**
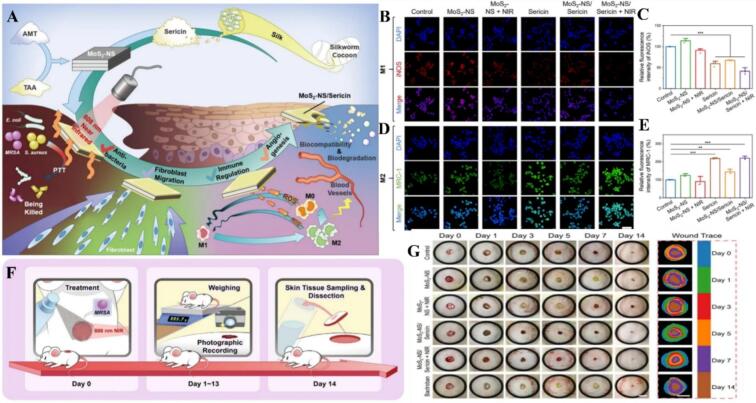
Sprayable 2D MoS₂/Silk Sericin bio-nanocomposite dressings with multifunctional and enhanced photothermal properties are used to improve infected wound healing. (A) Illustration of the fabrication of MoS2-NS/Sericin, which is endowed with photothermal conversion ability via 808 nm NIR irradiation. MoS2-NS/Sericin shows an antibacterial activity via PTT, fibroblast migration, and immune regulation as well as promotes angiogenesis, accelerates bacteria-infected wound healing. (B-E) ROS clearance and macrophage polarization modulation experiments of RAW 264.7 macrophages after co-incubation with MoS2-NS, MoS2-NS + NIR, sericin, MoS2-NS/Sericin, and MoS2-NS/Sericin + NIR. (B) Immunostaining of iNOS (red) and (D) CD206 (green) in RAW 264.7 macrophages after 24 h co-incubation. Scale bar = 50 μm. Quantification of relative fluorescence intensity of (C) iNOS and (E) CD206 using Image J (*n* = 3). Data are means ± S.E.M (colour figure online) (F) Protocol of the wound healing experiment on the bacteria (MRSA)-infected full-thickness skin model. (G) Representative photographs (scale bar = 1 cm) and closure traces (scale bar = 5 mm) of skin wounds treated with pure SPSS (control), MoS2-NS, MoS2-NS + NIR, MoS2-NS/Sericin, MoS2-NS/Sericin + NIR, and Bactroban on day 0, 1, 3, 5, 7 and 14. Reference (Qiu et al., 2024) with permission from Copyright © 2025, Advanced Science. (For interpretation of the references to colour in this figure legend, the reader is referred to the web version of this article.)

Molybdenum disulfide (MoS₂) has transitioned from its conventional roles in anti-friction materials, batteries, and biosensors to become a potent platform for modulating macrophage-driven disease pathologies (Dong et al., 2021; Du et al., 2024; Li et al., 2024a). Unlike traditional metal nanoparticles, MoS₂ nanostructures possess unique enzyme-like activities (nanozymes) and tunable surface chemistry that allow for sophisticated control over cellular phenotypes. In the treatment of traumatic spinal cord injury (SCI), for instance, MoS₂@PEG nanoflowers loaded with etanercept (ET) leverage high tissue penetration to inhibit pro-inflammatory M1 markers (TNF-α, CD86, iNOS) while concurrently upregulating anti-inflammatory M2 markers (Arg1, CD206, IL-10), thereby protecting motor neurons (Ge et al., 2021). This ability to resolve neuroinflammation is particularly critical in Alzheimer's disease (AD), where the brain's microglial balance is disrupted by amyloid-beta (Aβ) deposition and oxidative stress (Sun et al., 2019). Recent breakthroughs highlight how MoS₂ quantum dots modified with macrophage membranes (MoS₂QDs/MM) act as targeted antioxidants, eliminating ROS and resisting Aβ deposition under near-infrared (NIR) irradiation (Qi et al., 2024). Critical to this neuroprotection is the targeted delivery to mitochondria; TPP-MoS₂ nanozymes (and their modified variants) cross the blood-brain barrier to switch microglia from a neurotoxic M1 phenotype to a reparative M2 phenotype, facilitating the phagocytic clearance of Aβ aggregates and normalizing microglial dysfunction (Ren et al., 2020; Alomari et al., 2023). Beyond neuroprotection, the catalytic and photothermal properties of MoS₂ are harnessed to drive divergent macrophage responses in oncology and regenerative medicine. In cancer immunotherapy, 1T2H-MoS₂ nanozymes utilize enhanced peroxidase-like activity to repolarize TAMs toward an immunogenic M1-like phenotype, which directly kills cancer cells and amplifies the systemic immune response (Cui et al., 2024). Conversely, in the field of wound healing, MoS₂ is engineered to provide a sequential or synergistic response to infection and inflammation. For example, sericin-exfoliated MoS₂ nanosheets (MoS₂-NS/Sericin) combine photothermal antibacterial action with ROS scavenging to transform M1 macrophages into the M2 phenotype, thereby accelerating fibroblast migration and full-thickness skin recovery (Qiu et al., 2024). The multifunctional and enhanced photothermal properties of sprayable 2D MoS₂/silk sericin bio-nanocomposite dressings for infected wound healing are illustrated in Fig. 4. This “intelligent” temporal regulation is further exemplified by the MoS₂-ACF heterojunction structure (MAPS), which provides a dual-phase therapeutic window: early-stage NIR-induced photothermal effects drive M1 polarization to eradicate bacteria, while subsequent ROS scavenging triggers M2 reprogramming to resolve inflammation and stimulate cell proliferation (Mao et al., 2025). In conclusion, MoS₂ nanoparticles represent a versatile class of “smart” materials that integrate catalytic nanozyme activity with responsive photothermal properties. By selectively driving M1 polarization for tumor eradication and antibacterial action, or M2 polarization for neuroprotection and wound repair, MoS₂-based platforms offer a sophisticated approach to resolving complex inflammatory imbalances across diverse clinical applications (Table 3).

**Table 3:** MoS_2_NPs act on macrophages to treat various diseases.

Nanoparticle	Effect on Macrophages	Key Mechanism	Disease Application	References
MoS₂@PEG Nanoflowers	Inhibits M1 polarization, promotes M2 polarization	Delivers etanercept (ET), downregulates TNF-α, CD86, iNOS, upregulates Arg1, CD206, IL-10	Traumatic Spinal Cord Injury (SCI)	(Ge et al., 2021)
TPP-MoS₂ nanozymes	Switch microglia from M1 to M2 phenotype	Target mitochondria; cross blood-brain barrier; escape lysosomes; regulate cytokines	Alzheimer's Disease (AD)	(Ren et al., 2020)
TPP-MoS₂ quantum dots	Stimulate M1-to-M2 polarization of microglia	Inhibit Aβ accumulation; degrade Aβ deposits	Alzheimer's Disease (AD)	(Alomari et al., 2023)
1T2H-MoS₂ Nanozyme	Repolarizes TAMs to M1-like phenotype	Enhances peroxidase activity (≈12 times), generates hydroxyl radicals, directly kills cancer cells and stimulates pro-inflammatory cytokines	Breast Cancer	(Cui et al., 2024)
MoS₂-NS/Silk Fibroin Dressing	M1 → M2 polarization, promotes wound healing	Photothermal antibacterial (808 nm), scavenges ROS, enhances oxygen permeability and cell migration	Infected Wounds	(Qiu et al., 2024)
MoS₂-ACF Heterostructure (MAPS)	Early promotion of M1 (bactericidal), later transition to M2 (anti-inflammatory)	NIR photothermal effect induces M1, ROS scavenging ability reprograms to M2	Chronic Wounds	(Mao et al., 2025)

### 3.4 Titanium dioxide nanoparticles (TiO₂ NPs): Balancing Photocatalytic potential with Immunological Dynamics

**Fig. 5:**
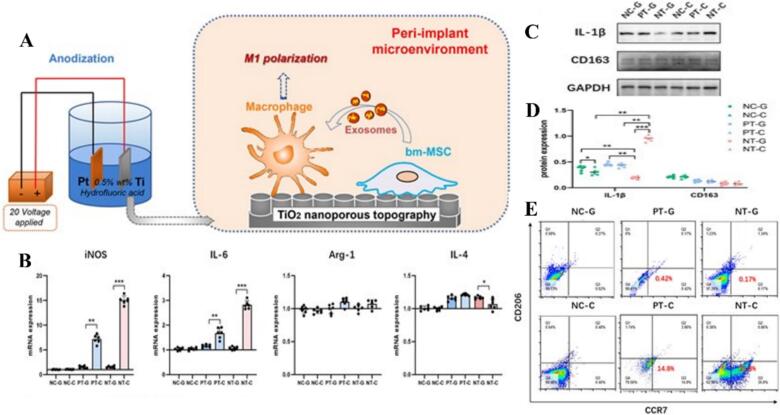
Unique modulation of macrophage polarization by TiO₂ nanoporous topography through MSC-derived exosomes. (A) Schematic illustration of the unique mechanism by which TiO₂ nanoporous topography regulates macrophage polarization via MSC-derived exosomes. (B-E)Effects of CM without exosomes for bm-MSCs induced by the different surface topographies of Ti specimens on macrophage polarization. The secretion of exosomes was blocked by GW4869 (or DMSO as control). (B) qRT-PCR analysis of mRNA expression of M1/M2 macrophage markers (M1: iNOS and IL-6; M2: Arg-1 and IL-4). (C) Western blot analysis of protein expression of the M1/M2 macrophage markers (M1: IL-1β; M2: CD163). (D) Semi-quantitative analysis of the protein expression level. (E) Flow cytometry analysis of M1/M2 macrophage markers (M1: CCR7; M2: CD206). NC-C, negative control with DMSO; PT-C, polished Ti with DMSO; NT-C, nanoporous Ti with DMSO; NC-G, negative control with GW4869; PT-G, polished Ti with GW4869; NT-G, nanoporous Ti with GW4869. Reference (Wang et al., 2023a) with permission from Copyright © 2025, Advanced Science.

Titanium dioxide nanoparticles (TiO₂ NPs) have established a significant presence in industrial sectors such as battery manufacturing, chemical sensing, and photocatalysis, yet their biological utility is increasingly defined by their complex and often concentration-dependent interactions with the immune system (Pillai et al., 2024; Cheng et al., 2024a; Bayles et al., 2022; Kose et al., 2021). In the context of orthopedic and dental applications, the surface topography and chemical doping of titanium-based materials are critical for favorable clinical outcomes. For instance, Bo Chen et al. demonstrated that zinc-doped TiO₂ nanotubes (TNTs) on titanium implants can create a pro-osteogenic environment by modulating macrophage functions; specifically, these surfaces promote the expression of M2 anti-inflammatory markers while moderately inhibiting M1 markers, thereby facilitating bone formation through a balanced immune response (Chen et al., 2020b). However, the immunomodulatory effects of TiO₂ are highly sensitive to physical parameters and delivery contexts. Studies examining varying sizes and concentrations of TiO₂ nanoparticles indicate that the magnitude of the inflammatory stimulus is highly dependent on these physical parameters. Specifically, higher concentrations effectively polarize macrophages toward the M1 phenotype by inducing the production of pro-inflammatory markers such as TNF-α and CD197 (Schoenenberger et al., 2016). Furthermore, the specific activation state and potential for disrupting immune balance are significantly influenced by the particle's size, where different dimensions can prime macrophages for varying degrees of susceptibility to secondary infections. While such M1 polarization is theoretically desirable in oncology to suppress growth-factor-secreting, anti-inflammatory M2 TAMs, the systemic implications of TiO₂-induced polarization present significant risks. Specifically, TiO₂ NPs can trigger M1 polarization in a TLR4-dependent manner, a process that potentially disrupts the homeostatic immune balance and increases patient susceptibility to secondary bacterial infections (Huang et al., 2017). This complexity is further highlighted by the discovery that under the influence of TiO₂, bone marrow mesenchymal stem cells (bm-MSCs) can exert adverse immunomodulatory effects; through exosome-mediated pathways, they may induce M1 polarization that negatively impacts the local osteogenic microenvironment around implants (Wang et al., 2023a), as shown in Fig. 5. In conclusion, titanium dioxide nanostructures serve as a double-edged sword in biomedical engineering. While zinc-doped TiO₂ nanotubes offer a promising route for regenerative bone repair through M2 induction, the inherent tendency of TiO₂ NPs to drive TLR4-dependent M1 polarization requires careful management to avoid chronic inflammation or compromised immune defense in clinical applications (Table 4).

**Table 4:** TiO₂NPs act on macrophages to treat various diseases.

Nanoparticle	Effect on Macrophages	Key Mechanism	Disease Application	References
Zn-doped TiO₂ Nanotubes (TNT)	Enhanced expression of M2 markers, suppression of M1 markers	Zn doping promotes M2 polarization of macrophages, establishing an osteogenic microenvironment	Surface modification of bone implants	(Chen et al., 2020b)
High-concentration TiO₂ Nanoparticles	Promotes M1 polarization (↑TNF-α, ↑CD197)	High concentrations of TiO₂ NPs stimulate macrophages to secrete pro-inflammatory factors	Immune regulation/Inflammatory response studies	(Schoenenberger et al., 2016)
TiO₂ NPs (TLR4-dependent pathway)	Promotes M1 polarization, potentially disrupts immune balance	Activates M1 macrophages via the TLR4 signaling pathway, increases susceptibility to bacterial infections	Infection and immune regulation	(Huang et al., 2017)
TiO₂ Nanotubes (80–100 nm)	Induces M1 polarization of macrophages	BM-MSCs promote M1 polarization through exosome-mediated paracrine effects on nanoporous TiO₂ surfaces	Regulation of osteogenic microenvironment for bone implants	(Wang et al., 2023a)

### 3.5 Zinc-based nanoparticles: strategic dual-action platforms for oncology and immunotherapy

**Fig. 6:**
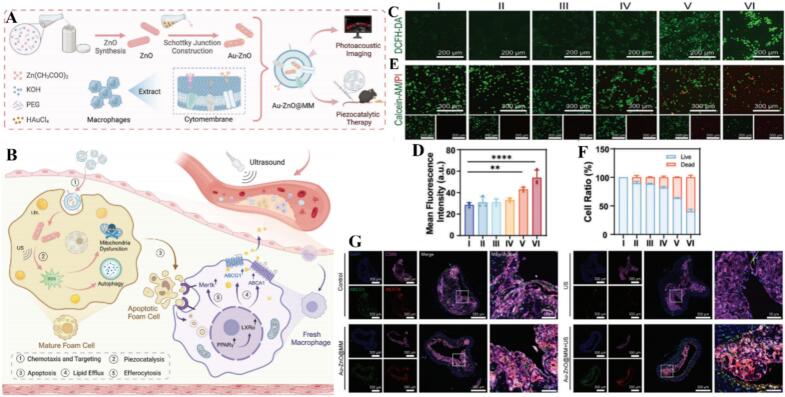
Membrane-camouflaged Au-ZnO nanorods enter foamy macrophages, ultrasound-triggered ROS turns them into apoptotic debris, and the debris trains fresh macrophages to export cholesterol and clean the plaque. (A,B) Schematic diagram showing the fabrication process of biomimetic Au-ZnO@MM Schottky junction and its mechanism of piezocatalytic atherosclerosis therapy. (A) The synthetic route and application of Au-ZnO@MM Schottky junction including photoacoustic imaging and piezocatalytic theranostics. (B) The biomimetic Trojan horse strategy mediated by Au-ZnO@MM Schottky junction for atherosclerotic treatment. (C—F)Selective targeting and effective elimination of foamy macrophages by biomimetic Au-ZnO@MM Schottky junction in vitro. (C,D) Representative fluorescence images (C) and corresponding quantitative analysis (D) of intracellular ROS stained by DCFH-DA(*n* = 3). (E,F) Representative fluorescence images (E) and quantitative analysis (F) of live-dead staining of foam cells with various treatments (n = 3). (I) Immunofluorescence assay and colocalization of aortic arch sections showing cholesterol efflux and efferocytosis-related proteins on macrophages after various treatments (blue, nucleus stained with DAPI; pink, Cy5-labeled CD68; green, FITC-labeled ABCG1; red, Cy3-labeled Mertk). Reference (Cheng et al., 2024b) with permission from Copyright © 2025, Advanced Science. (For interpretation of the references to colour in this figure legend, the reader is referred to the web version of this article.)

Zinc-based nanoparticles, particularly Zinc Oxide (ZnONPs) and Zinc Sulfide (mZnS), represent a sophisticated class of multifunctional nanomedicines that bridge the gap between direct cytotoxicity and targeted immunomodulation. A critical analysis of recent literature suggests that the therapeutic efficacy of ZnONPs stems not only from their ability to serve as drug carriers but also from their intrinsic capacity to reprogram the tumor and inflammatory microenvironments. For instance, Jiao Wang et al. demonstrated that DOX@ZnO NPs can effectively penetrate complex 3D tumor architectures to target multidrug-resistant (MDR) cells; notably, the ZnONPs themselves downregulate the cancer stem cell marker CD44, thereby reducing “stemness” and sensitizing the tumor to chemotherapy (Wang et al., 2017). This direct antitumor action is synergistically coupled with a unique protective effect on the immune system: unlike free chemotherapeutics, ZnO NPs shield macrophages from drug-induced toxicity while simultaneously driving their polarization toward a pro-immunogenic M1-like phenotype, effectively turning the macrophage-conditioned medium into a potent agent for cancer cell apoptosis (Wang et al., 2017). However, the application of ZnONPs requires a nuanced understanding of dose-dependency and biological context, as their interaction with macrophages is highly sensitive to concentration. As observed by SiJia Geng, the interaction between ZnONPs and macrophages is characterized by a nuanced, dose-dependent biological response. As observed in functional assays, low doses of ZnONPs can trigger beneficial autophagy by increasing pro-inflammatory cytokines, which contributes to overall immune regulation. However, shifting to high doses can dramatically alter macrophage behavior, driving the cellular response toward ferroptosis. This dose-mediated switch highlights that the therapeutic or cytotoxic outcome is not intrinsic to the material alone but is dictated by the precise dose metrics employed, a mechanism that could be harnessed for future antibacterial strategies (Geng et al., 2023). This versatility extends beyond oxides to other zinc chalcogenides; for example, mesocrystalline ZnS nanoparticles (mZnS) leverage the tumor microenvironment to generate H_2_ and O_2_, providing the necessary metabolic cues to drive M2-to-M1 macrophage repolarization and subsequent CD8 T-cell activation (Xia et al., 2023). Furthermore, the biocompatibility and regulatory potential of these materials are enhanced through biomimetic strategies, such as the Au-ZnO@MM “Trojan horse” system. By coating particles in macrophage membranes, researchers have successfully redirected the innate immune response to alleviate atherosclerosis progression, underscoring that zinc-based nanostructures are no longer just passive vehicles but active orchestrators of macrophage fate across oncological and cardiovascular pathologies (Cheng et al., 2024b). For the mechanistic illustration of membrane-camouflaged Au-ZnO nanorods entering foamy macrophages, ultrasound-triggered ROS generation, and debris-mediated training of fresh macrophages for cholesterol export and plaque clearance, as shown in Fig. 6. In conclusion, zinc-based nanoparticles offer a multi-targeted therapeutic approach by combining direct tumor sensitization with precise macrophage reprogramming. Their ability to switch between inducing protective autophagy or therapeutic cell death, while simultaneously promoting an M1-mediated immune response, establishes them as highly adaptive platforms for overcoming drug resistance and chronic inflammatory imbalances (Table 5).

**Table 5:** ZnNPs act on macrophages to treat various diseases.

Nanoparticle	Effect on Macrophages	Key Mechanism	Disease Application	References
DOX@ZnO NPs	Promote M1-like polarization of macrophages, protect macrophages from DOX toxicity	Downregulate CSC marker CD44, reduce cancer cell stemness; enhance pro-inflammatory cytokine secretion by macrophages, promote cancer cell apoptosis	Cancer (especially MDR cancer)	(Wang et al., 2017)
Low-dose ZnO NPs	Inhibit M1-like polarization of macrophages	Enhance BCG-induced immune response through autophagy mechanism	Antibacterial/Immune regulation	(Geng et al., 2023)
Mesocrystalline ZnS (mZnS)	Induce repolarization of intratumoral macrophages from M2 to M1	Sonolysis of water to produce H₂ and O₂, alleviate tumor hypoxia and activate CD8^+^ T cells	Deep tumor immunotherapy	(Xia et al., 2023)
Au-ZnO@MM Biomimetic Nanoparticles	Regulate macrophage phenotype (M1/M2 not specified), alleviate inflammation	Macrophage membrane (MM) coating enhances targeting, Schottky junction (Au-ZnO) promotes anti-inflammatory effects	Atherosclerosis	(Cheng et al., 2024b)

### 3.6 Manganese Dioxide (MnO₂) nanoparticles: catalytic oxygenation and redox regulation of macrophage phenotypes

**Fig. 7:**
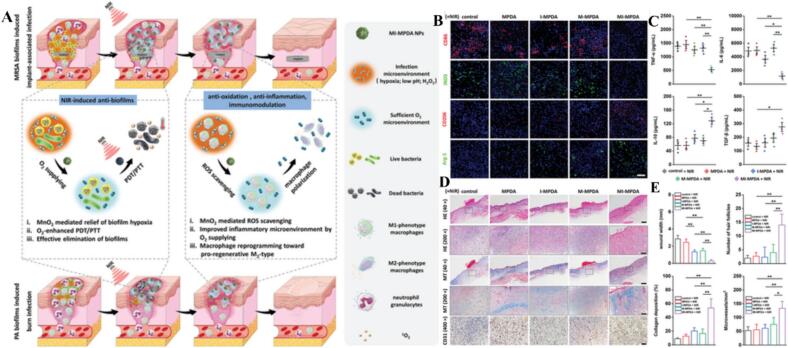
A bio-responsive, oxygen-self-supplying photo-therapeutic nanocomposite eradicates drug-resistant bacterial biofilms and their associated inflammation. (A)Schematic illustration of NIR-irradiated MI-MPDA for treating MRSA biofilm-induced subcutaneous implant-associated infection and PA biofilm-induced burn infection. (B) The representative images of immunofluorescent staining of the surface biomarkers of M1 (CD86 and iNOS) and M2 (CD206 and Arg-1) macrophages in the wound on day 14 days postoperatively (scale bar: 50 μm). (C) In vivo pro-inflammatory and anti-inflammatory cytokines measurements (*n* = 3). (D) Representative images of HE staining, Masson's trichrome staining, and CD31-immunohistochemical staining (Scale bars: 500 μm for 40×, 100 μm for 200×, and 50 μm for 400×). (E) Semi-quantitative analysis of the wound width, hair follicles number, collage deposition percentage, and microvessels density. Representative images were taken from three independent samples (n = 3). Reference (Yuan et al., 2023) with permission from Copyright © 2025, Advanced Science.

Manganese dioxide (MnO₂) nanoparticles have emerged as a versatile class of “smart” nanomedicines, distinguished by their unique ability to catalyze the decomposition of endogenous hydrogen peroxide (H_2_O_2_) into oxygen (O_2_) while simultaneously regulating the redox microenvironment. In the field of oncology, MnO₂-based platforms are strategically employed to overcome the immunosuppressive nature of the tumor microenvironment (TME) by driving the repolarization of TAMs from a pro-tumor M2 phenotype to an anti-tumor M1 phenotype. For instance, Lijuan Chong et al. utilized hollow mesoporous manganese dioxide nanoparticles (HMMDN-Met@PM) to achieve this transition, effectively inhibiting tumor growth through targeted metabolic and phenotypic shift (Chong et al., 2023). This immunogenic reprogramming is often synergized with external physical therapies; hyaluronic acid-modified SAMMH, HA-integrated MnO₂ nanosheets, and MnO₂-encapsulated liposomal Lipo Zol/IR NPs all leverage sonodynamic or photodynamic effects to alleviate hypoxia via O_2_ production, thereby enhancing the M1-to-M2 conversion and amplifying anti-tumor immunity (Liu et al., 2024b; Ai et al., 2018; Jian et al., 2022). Advancing this paradigm, biomimetic nanoplatforms integrating multimodal imaging with synergistic phototherapy have been developed. A representative system employs hollow mesoporous MnO₂ (HMnO₂) functionalized with poly(allylamine hydrochloride) to achieve positive surface charge, enabling efficient loading of the photosensitizer indocyanine green (ICG) through counterion aggregation. The resulting nanoparticles are subsequently encapsulated with HeLa cell membranes to produce M-HMnO₂@ICG, which combines tumor-homing targeting with immune evasion capabilities. In this architecture, HMnO₂ responsively degrades in the TME to release ICG, self-generates O₂ to sensitize ICG-mediated photodynamic therapy (PDT), and consumes glutathione (GSH) to amplify oxidative stress, thereby integrating chemodynamic therapy (CDT) with PDT. Concurrently, accumulated ICG enables synergistic PDT/photothermal therapy (PTT) under single-laser irradiation, where the hyperthermia generated by PTT further enhances CDT kinetics. This nanosystem thus achieves full PTT/PDT/CDT synergy, with Mn^2+^ serving as a magnetic resonance imaging contrast agent and ICG providing near-infrared fluorescence and photothermal imaging for real-time treatment guidance (Wang et al., 2024c). Furthermore, targeted delivery systems such as the BSA-MnO₂-SAL (BMS) nanoparticles for glioblastoma and hpMNPs which upregulate nitric oxide production demonstrate how surface modification can refine the immunological impact of MnO₂ (Liang et al., 2021; Lim et al., 2022). The incorporation of catalytic MnO₂ into multi-component modulators, such as the IPI549-combined nanomodulator or tetrahedral DNA nanostructures (TDNs) combined with Mn^2+^, further illustrates the transition toward highly integrated systems that reshape the TME for improved therapeutic outcomes (Liang et al., 2022; Yu et al., 2019). Conversely, in non-oncological inflammatory pathologies, MnO₂ nanoparticles are engineered to function as potent antioxidants and oxygenators to resolve chronic inflammation and promote tissue repair. In these contexts, the objective shifts toward inhibiting M1 polarization and fostering a reparative M2 environment. Specifically, MnO_2_ nanoparticles function as intelligent nanozymes that modulate the MAPK signaling cascade through redox buffering. By alleviating oxidative stress and suppressing the NOX4 p38 MAPK pathway, this intervention prevents the sustained activation of stress-activated protein kinases, thereby steering macrophages away from a chronic pro-inflammatory state and toward a phenotype conducive to tissue healing. For musculoskeletal applications, MnO₂@TMNP prevents M1-mediated disc degeneration and pain by scavenging reactive oxygen species (ROS), while the HP@Mn barrier membrane regulates the ROS microenvironment to accelerate bone repair (Yang et al., 2024; Liu et al., 2023). Similarly, the L-MNS-CMDA hydrogel utilizes MnO₂ nanosheets to inhibit inflammatory polarization, favoring cartilage regeneration (Zhao et al., 2025c). This oxygen-supplying and ROS-scavenging capability is particularly vital in compromised microenvironments; for example, the titanium implant-integrated MCM-Ti system alleviates hypoxia to drive M2 polarization in diabetic conditions, while the yeast cell wall-encapsulated YMD@MPDA nanozyme complex protects against colitis by resolving oxidative stress (Wu et al., 2024; Zhang et al., 2025b). Finally, the MI-MPDA nanocomposite exemplifies the dual-utility of MnO₂ as both an antibacterial agent and a pro-healing modulator, catalyzing O_2_ supply to treat biofilm infections while steering macrophages toward the M2 state (Yuan et al., 2023), with the bio-responsive, oxygen-self-supplying photo-therapeutic mechanism for eradicating drug-resistant bacterial biofilms and resolving associated inflammation visually summarized in Fig. 7. Collectively, these studies underscore that MnO₂ is not a static carrier but a dynamic orchestrator of macrophage fate, capable of either igniting or quenching inflammation depending on the catalytic requirements of the disease site (Table 6).

**Table 6:** MnO₂NPs act on macrophages to treat various diseases.

Nanoparticle	Effect on Macrophages	Key Mechanism	Disease Application	References
HMMDN-Met@PM	M2 → M1 repolarization (CD206↓, Arg-1↓, IL-10↓; CD80↑, TNF-α↑, iNOS↑)	M2pep targets delivery of metformin to M2-TAMs, catalyzes H₂O₂ decomposition to relieve hypoxia, upregulates pro-inflammatory factors	Tumor immunotherapy	(Chong et al., 2023)
SAMMH (HA-MnO₂)	M2 → M1 repolarization, depletion of M2-TAMs	Dual targeting (HA-CD44 binding), sonodynamic therapy (SDT) combined with chemodynamic therapy (CDT), ROS generation and immune microenvironment remodeling	Tumor (combined sonodynamic therapy)	(Liu et al., 2024b)
MnO₂@TMNP	Inhibits M1 polarization, scavenges ROS	Encapsulated with TrkA-overexpressing macrophage membranes, scavenges ROS to block M1 polarization, alleviates inflammatory microenvironment	Intervertebral disc degeneration/pain	(Yang et al., 2024)
M-HMnO₂@ICG	M2 → M1 repolarization	HeLa cell membrane encapsulation enables homologous targeting and immune evasion; HMnO₂ catalyzes H₂O₂ decomposition to relieve hypoxia and consumes GSH to amplify oxidative stress; synergistic PTT/PDT/CDT under single NIR laser irradiation, where PTT-enhanced hyperthermia accelerates CDT kinetics; Mn^2+^ enables MRI guidance	Cervical cancer (synergistic phototherapy)	(Wang et al., 2024c)
BMS (BSA-MnO₂-SAL)	M2 → M1 repolarization	Acid/H₂O₂-responsive release of salinomycin (SAL), Mn^2+^ enhances STING pathway activation, synergistically upregulates pro-inflammatory factors	Glioblastoma (GBM)	(Liang et al., 2021)
TDN-MnO₂ complex	Promotes M1 polarization (IFN-β↑, iNOS↑)	Tetrahedral DNA nanostructures (TDN) activate the STING pathway, Mn^2+^ synergistically enhances immune response, reduces cytotoxicity	Tumor immunotherapy	(Liang et al., 2022)


Nanoparticle	Effect on Macrophages	Key Mechanism	Disease Application	References
HA-MnO₂ nanosheets	M2 → M1 repolarization	Degradation of H₂O₂ to produce oxygen enhances PDT efficiency, HA targets and synergistically reprograms TAMs	Tumor (photodynamic therapy)	(Ai et al., 2018)
hpMNPs (HA/PLR-MnO₂)	M2 → M1 repolarization, upregulates NO	PLR coating enhances immunomodulation, NO-dependent anticancer effects	Tumor immunotherapy	(Lim et al., 2022)
HP@Mn membrane (HAp NWs/MnO₂)	Inhibits M1 polarization, alleviates inflammation	MnO₂ catalyzes the degradation of H₂O₂ to relieve oxidative stress, improves bone repair microenvironment	Bone defect repair	(Liu et al., 2023)
MnO₂-IPI549 nanomodulator	M2 → M1 repolarization, enhances T cell infiltration	Relieves hypoxia (MnO₂ oxygen generation) combined with PI3Kγ inhibition (IPI549), downregulates PD-L1, reshapes tumor immune microenvironment (TIME)	Tumor immunotherapy combination	(Yu et al., 2019)
Lipo Zol/IR NPs (MnO₂)	M2 → M1 repolarization	MnO₂ catalyzes H₂O₂ to produce oxygen enhancing PDT, zoledronic acid (Zol) induces TAMs repolarization, IR780 photodynamic activation of immunity	Breast cancer photodynamic immunotherapy	(Jian et al., 2022)
MCM-Ti (MnO₂-CO@MPDA)	Drives M2 polarization (anti-inflammatory)	MnO₂ scavenges ROS and relieves hypoxia, CO gas regulates macrophage phenotype	Diabetic bone implant	(Wu et al., 2024)
L-MNS-CMDA hydrogel	Inhibits M1, promotes M2 polarization	MnO₂ decomposes H₂O₂ to relieve oxidative stress, catechol groups synergistically regulate immunity	Cartilage defect repair	(Zhao et al., 2025c)


Nanoparticle	Effect on Macrophages	Key Mechanism	Disease Application	References
YMD@MPDA (YCW-MnO₂)	M1↓, M2↑	MnO₂ scavenges ROS and triggers H₂S release, regulates NOX4/p38 MAPK pathway, restores mitochondrial function	Colitis (oral treatment)	(Zhang et al., 2025b)
MI-MPDA (MnO₂-ICG)	M1↓, M2↑ (HIF-1α↓)	MnO₂ catalyzes H₂O₂ to supply oxygen enhancing phototherapy, inhibits HIF-1α pathway to reprogram macrophages	Biofilm infection/inflammatory diseases	(Yuan et al., 2023)

### 3.7 Iron Oxide Nanoparticles (Fe₃O₄ NPs): Magnetic Targeting and Phenotypic Reprogramming in Immunotherapy

**Fig. 8:**
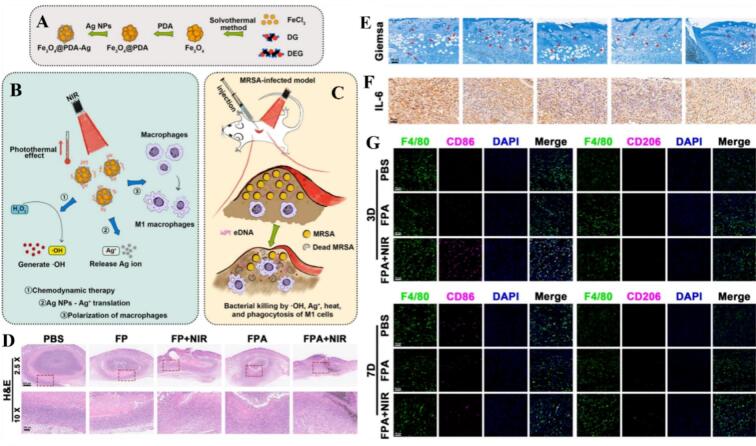
Infection microenvironment-triggered nanoparticles eradicate MRSA by thermally amplified chemodynamic therapy and M1 macrophage. (A-C) Graphical abstract: Infection-microenvironment-responsive nanoparticles eradicate MRSA through thermally amplified chemodynamic therapy and M1 macrophage reprogramming. (D-G)Histopathological evaluation of the skin. (D) Representative images of H&E staining. (E) Representative images of Giemsa staining: circular and darkly stained structures indicate bacteria; red arrows indicate remaining bacteria. (F) Representative images of IHC staining for IL-6. (G) Results of labeling different subtypes of cells with IF staining after collecting skin samples on days 3 and 7 after subcutaneous infection of mice treated with nanoparticles. Where co-localization of three signals with F 4/80 (green), CD 86 (red), and DAPI (blue) indicates M1-like cells, and co-localization of three signals with F 4/80, CD 206, and DAPI indicates M2-like cells. Reference (Hong et al., 2024) with permission from Copyright © 2025, Advanced Science. (For interpretation of the references to colour in this figure legend, the reader is referred to the web version of this article.)

Iron oxide nanoparticles (Fe₃O₄ NPs) are established clinical tools, primarily utilized as magnetic resonance imaging (MRI) contrast agents and targeted drug carriers due to their superparamagnetic properties and biocompatibility. Recent research has unveiled their intrinsic immunomodulatory potential, specifically their ability to serve as catalytic or magnetic triggers for macrophage repolarization. In the field of oncology, a dominant therapeutic strategy involves the conversion of immunosuppressive M2 TAMs into pro-inflammatory M1 phenotypes to ignite an anti-tumor response. This transition can be achieved through structural hybrids, such as Fe₃O₄/single-walled carbon nanotube (Fe₃O₄-SWCNT) composites that activate systemic tumor immunity (Zhou et al., 2022; Fu et al., 2020; Zhang et al., 2023b). Furthermore, Fe₃O₄ NPs are frequently functionalized with targeting ligands or agonists to refine this process; for instance, doxorubicin and hyaluronic acid-loaded Fe₃O₄ NPs, and biomimetic magnetic nanocarriers co-loaded with the TLR7 agonist imiquimod (R837), achieve M1 polarization through IRF5 signaling activation (Gong et al., 2019; Liu et al., 2020). The precision of these interventions is often guided by imaging, as seen in superparamagnetic iron oxide nanoparticles (SPIO) modified with M2-targeting peptides for MRI-guided hyperthermia, and anti-CD206 antibody-modified Fe₃O₄-PLGA nanoparticles that directly reverse the TAM phenotype (Wang et al., 2021b; Zhou et al., 2020). The integration of Fe₃O₄ NPs into responsive delivery systems such as ultrasound-sensitive nanobubbles—represents a frontier in spatial and temporal immune control. Systems like FA-PFNB-SIRPα siRNA and MPFS@NDs utilize the combination of Fe3O4 and gene silencing to reverse Siglec-G/SIRPα-mediated inhibition, thereby enhancing macrophage phagocytosis (Li et al., 2024b; Li et al., 2024c) Notably, the therapeutic efficacy of iron-based MNPs in tumor immunotherapy is often tied to their ability to disrupt immunosuppressive signaling. A representative strategy involves the use of folic acid-modified iron oxide nanobubbles co-loaded with STAT3 siRNA. These systems not only leverage magnetic targeting but also achieve precise silencing of the JAK/STAT3 signaling pathway. By preventing STAT3 phosphorylation, these nanocarriers effectively reverse the M2-like state of TAMs, restoring their ability to promote anti-tumor T cell responses (Yin et al., 2024). Moreover, bimetallic hybrids like the iron‑manganese Fe₃O₄@C/MnO₂-PGEA nanohybrid broaden this impact by promoting dendritic cell maturation alongside macrophage repolarization (Zhao et al., 2022). Biomimetic strategies, such as the macrophage membrane-coated hollow Fe₃O₄ nanoparticles (FBN@M) co-loaded with BMS-202, further optimize therapy by simultaneously reprogramming TAMs and blocking the PD-1/PD-L1 checkpoint (Kang et al., 2024). In infectious and complex microenvironments, Fe₃O₄@PDA-Ag nanoparticles synergize ROS generation with Ag^+^ release to kill bacteria while recruiting innate immune clearance via M1 polarization, the mechanism illustrated in Fig. 8 (Hong et al., 2024). Conversely, the versatility of Fe₃O₄ allows for the promotion of reparative M2 polarization when the therapeutic goal shifts toward tissue regeneration or bone repair. In these contexts, magnetic targeting and sustained release are paramount; bFGF-loaded Fe₃O₄ nanoparticles (bFGF-HDC@Fe₃O₄) utilize external magnetic field stimulation to accelerate wound healing, while the BCL@MMSNPs-SS-CD-NW composite leverages glutathione-responsive release to foster an M2 environment for bone repair (Wu et al., 2021; Zhou et al., 2021). Innovative scaffolds like the GdPO₄/CS/Fe₃O₄ platform provide a dual-phase solution by combining photothermal ablation of residual tumor cells with subsequent M2-mediated bone regeneration (Zhao et al., 2020). Finally, the ultrasound-responsive Fe₃O₄/HA/Ber-LA hydrogel exemplifies a “smart” immunomodulatory approach, utilizing sonodynamic ROS and nitric oxide (NO) generation to navigate the complex transition from a pro-inflammatory to an anti-inflammatory state (Wang et al., 2024d). Collectively, these studies demonstrate that Fe₃O₄ nanoparticles are not merely passive carriers but dynamic regulators of the macrophage rheostat, capable of either intensifying or resolving inflammation based on the specific engineering of their magnetic and chemical interface (Table 7).

**Table 7:** Fe₃O₄NPs act on macrophages to treat various diseases.

Nanoparticle	Effect on Macrophages	Key Mechanism	Disease Application	References
Fe₃O₄-SWCNT@M1	M2 → M1 repolarization (activating tumor immune response)	Nanocomposite directly delivers M1 phenotype signals, reshapes tumor microenvironment	Tumor immunotherapy	(Zhang et al., 2023b)
Fe₃O₄-HA-DOX	M2 → M1 repolarization (synergistic tumor killing with DOX)	HA targets TAMs, Fe₃O₄ activates IRF5 pathway, DOX directly kills tumor cells	Tumor combination therapy	(Gong et al., 2019)
PIR@M (Fe₃O₄-R837@macrophage membrane)	M2 → M1 repolarization (CD86↑, TNF-α↑)	TLR7 agonist (R837) combined with Fe₃O₄ activates IRF5 pathway, macrophage membrane enhances targeting	Breast cancer immunotherapy	(Liu et al., 2020)
M2pep-SPIO	Targets M2-TAMs (MRI imaging-guided MHT)	Superparamagnetic iron oxide (SPIO) for magnetic hyperthermia, M2pep targeting peptide specifically recognizes M2 macrophages	Breast cancer diagnosis and treatment	(Wang et al., 2021b)
FCMP (Fe₃O₄@C/MnO₂-PGEA)	M2 → M1 repolarization, promotes DC maturation	Fe/Mn bimetallic synergy activates immune response, reverses immunosuppressive microenvironment	Tumor immunotherapy	(Zhao et al., 2022)
FA-PFNB-STAT3 siRNA/Fe₃O₄	M2 → M1 repolarization (IRF5↑), inhibits STAT3 pathway	Fe₃O₄ activates IRF5, siRNA silences STAT3 to block M2 polarization, folic acid targets delivery	Non-small cell lung cancer (NSCLC)	(Li et al., 2024b)
FA-PFNB-SIRPα siRNA/Fe₃O₄	M2 → M1 repolarization, enhances phagocytic function	Fe₃O₄ as immune adjuvant, siRNA silences SIRPα signal, ultrasound-triggered release	augmented non-small cell lung cancer immunotherapy	(Li et al., 2024c)


Nanoparticle	Effect on Macrophages	Key Mechanism	Disease Application	References
MPFS@NDs (Siglec-G siRNA/Fe₃O₄)	M2 → M1 repolarization, blocks CD24/Siglec-G signaling	Fe₃O₄ promotes M1 polarization, siRNA relieves immune suppression, mannose modification enhances targeting	Tumor immunotherapy	(Yin et al., 2024)
bFGF-HDC@Fe₃O₄	Promotes M2 polarization (anti-inflammatory phenotype)	Magnetic field (eMF) enhances bFGF release, induces M2 polarization to promote tissue repair	Wound healing	(Wu et al., 2021)
Anti-CD206-Fe₃O₄-PLGA	M2 → M1 repolarization (CD86↑)	Anti-CD206 antibody targets M2-TAMs, Fe₃O₄ upregulates M1 markers	Tumor immunotherapy	(Zhou et al., 2020)
Fe₃O₄@PDA-Ag	Promotes M1 polarization (bactericidal immunity)	Fe₃O₄ catalyzes ROS generation, Ag^+^ kills bacteria, photothermal synergy enhances immune response	MRSA infection	(Hong et al., 2024)
FBN@M (Fe₃O₄/BMS-202/NaHCO₃)	M2 → M1 repolarization, blocks PD-1/PD-L1	Fe₃O₄ reprograms TAMs, pH-responsive release of BMS-202, macrophage membrane camouflage	Tumor immunotherapy combination	(Kang et al., 2024)
Fe₃O₄/HA/Ber-LA hydrogel	Dynamic regulation (M1↑ during sterilization, M2↑ during repair)	Sonodynamic generation of ROS (M1 polarization), Berberine (Ber) induces M2 later	Infection-wound healing integration	(Wang et al., 2024d)
BCL@MMSNPs-SS-CD-NW (Fe₃O₄)	Promotes M2 polarization (anti-inflammatory)	Magnetic field targets delivery of BCL drugs, glutathione-responsive release, induces M2 phenotype	Fracture repair	(Zhou et al., 2021)
GdPO₄/CS/Fe₃O₄ scaffold	Promotes M2 polarization (pro-angiogenic)	Fe₃O₄ photothermal ablation of tumor, Gd^3+^ induces M2 polarization to promote bone regeneration	Postoperative tumor bone regeneration	(Zhao et al., 2020)

### 3.8 Calcium Carbonate (CaCO₃) Nanoparticles: pH-responsive Modulation of the Acidic Microenvironment

**Fig. 9:**
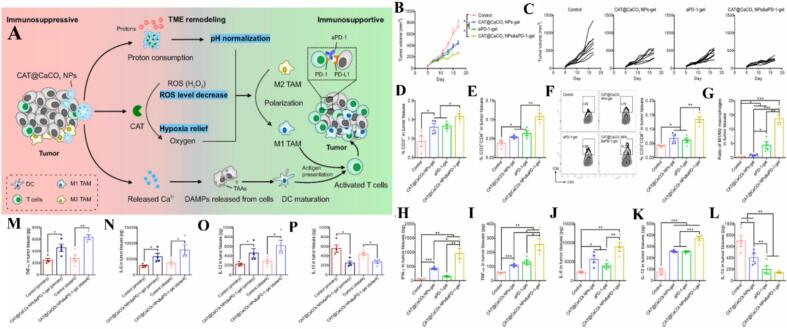
Tumor microenvironment modulation by CAT@CaCO₃NPs promotes M2-to-M1 macrophage polarization and enhances antitumor immunity. (A) Mechanistic diagram of CAT@CaCO₃NPs regulating macrophage polarization for antitumor immunotherapy. (B-L)In vivo antitumoral effect of CAT@CaCO3NPs combined with aPD-1. (B) Average and (C) individual tumor growth kinetics in different groups (*n* = 7–10). (D) Percentages of TIL (CD3+) and (E) CD4+ T cells (CD4 + CD3+) in tumors. (F) Flow cytometry analysis of CD8+ T cells (CD8+ in CD3+) in tumors. (G) Ratio of M1 (F4/80 + CD80+) to M2 macrophages (F4/80 + CD206+). Levels of (H) IFN-γ, (I) TNF-α, (J) IL-6, (K) IL-12, and (L) IL-10 in 50 mg tumor tissues. (M-P) In vivo antitumoral effect of CAT@CaCO3NPs combined with aPD-1 on the distant tumor model. Levels of (M) TNF-α, (N) IL-6, (O) IL-12, and (P) IL-10 in 50 mg tumor tissues. Reference (Fang et al., 2024) with permission from Copyright © 2025, Advanced Science.

Calcium carbonate (CaCO₃) nanoparticles have gained significant attention as smart, stimuli-responsive platforms due to their inherent acid-sensitivity and biocompatibility, particularly in overcoming the physiological barriers of the tumor microenvironment (TME). The TME is defined by significant immunosuppressive properties, characterized by physicochemical abnormalities such as acidity, hypoxia, and elevated reactive oxygen species (ROS) resulting from aberrant tumor metabolism (Ochoa de Olza et al., 2020; Wang et al., 2023b). This hostile environment actively suppresses the differentiation and antigen-presenting capabilities of dendritic cells, impairs T-cell infiltration and survival, and drives the polarization of TAMs toward the immunosuppressive M2 phenotype. Recent research demonstrates that CaCO₃-based nanostructures can be engineered to reverse this suppression by neutralizing acidity or delivering oxygen-generating payloads. For instance, Tianxu Fang et al. developed catalase-loaded calcium carbonate nanoparticles (CAT@CaCO₃NPs) that effectively decompose endogenous hydrogen peroxide into oxygen; this relief of tumor hypoxia and acidity serves as a biochemical trigger to promote M2-to-M1 macrophage polarization, thereby enhancing the overall efficacy of antitumor immunity, with the underlying mechanism of CAT@CaCO₃NPs-mediated tumor microenvironment modulation, M2-to-M1 macrophage reprogramming, and consequent antitumor immune enhancement visually summarized in Fig. 9 (Fang et al., 2024). Building upon this responsiveness, the integration of secondary minerals or phototherapeutic agents allows for a multi-modal assault on the TME. Mengyu Chang et al. created a pH/H₂S dual-responsive Cu₂O@CaCO₃ nanostructure that undergoes in situ conversion into Cu₃₁S₁₆ nanocrystals; this transformation provides photothermal and photodynamic activity that synergistically reprograms M2 macrophages to the M1 state, significantly inhibiting colorectal cancer recurrence (Chang et al., 2020). Similarly, the pH-responsive VMMFTTC nanoparticles utilize the acidic TME to trigger the release of functional molecules that induce localized oxidative stress, facilitating the M2-to-M1 transformation and activating a systemic antitumor immune response (Chang et al., 2024). Beyond oncology, the pH-responsiveness and mineral-donating properties of CaCO₃ are equally effective in regenerative medicine, where the therapeutic objective shifts toward resolving inflammation and promoting biomineralization. In cranial bone repair, Yan Zheng et al. designed a core-shell nanofiber implant incorporating CaCO₃@CurNPs and black phosphorus nanosheets (Zheng et al., 2024). Unlike the pro-inflammatory objectives in cancer therapy, this system leverages the degradation of CaCO₃ to provide calcium ions for bone growth while simultaneously steering macrophages from an M1 to a reparative M2 phenotype. Collectively, these studies illustrate that CaCO₃ nanoparticles are not merely passive carriers but dynamic modulators that can be fine-tuned to either ignite or quench inflammatory responses based on the specific requirements of the disease microenvironment (Table 8).

**Table 8:** CaCO₃NPs act on macrophages to treat various diseases.

Nanoparticle	Effect on Macrophages	Key Mechanism	Disease Application	References
CAT@CaCO₃ NPs	M2 → M1 repolarization (enhanced antigen presentation)	Catalase (CAT) decomposes H₂O₂ to relieve oxidative stress, CaCO₃ neutralizes acidic TME, reversing immunosuppression	Broad-spectrum antitumor immunotherapy	(Fang et al., 2024)
Cu₂O@CaCO₃ (→Cu₃₁S₁₆)	M2 → M1 repolarization (synergistic with CD47 antibody)	pH/H₂S responsive transformation to Cu₃₁S₁₆ nanocrystals, photothermal/photodynamic induction of thermal/oxidative stress, reprogramming of TAMs	Postoperative treatment of colorectal cancer	(Chang et al., 2020)
VMMFTTC (CaCO₃-TPP/THT/VK₂/MA)	M2 → M1 repolarization (cascade of oxidative stress)	pH-responsive release of THT (Fenton reaction), VK₂/MA (enhanced ROS), TPP targets mitochondria, multiple pro-inflammatory signals activated	Tumor immunotherapy combination	(Chang et al., 2024)
CaCO₃@CurNPs/black phosphorus nanosheets	M1 → M2 transformation (anti-inflammatory)	Curcumin (Cur) induces M2 polarization, Ca^2+^/PO₄^3−^ release promotes bone mineralization, black phosphorus enhances osteogenesis	Cranial bone defect repair	(Zheng et al., 2024)

### 3.9 Diverse metal-based nanostructures: synergistic approaches to chronic inflammatory control

Beyond the widely studied gold and silver platforms, a broader spectrum of metal-based nanostructures including cobalt, cerium, and ruthenium has demonstrated significant potential in modulating macrophage plasticity to treat complex inflammatory diseases. A common thread in recent research is the use of high-porosity or multi-metal frameworks to achieve sustained drug release and microenvironmental regulation. For instance, Yi Sun et al. leveraged the structural advantages of a cobalt-based MO to develop FZ@ZIF-67 nanoparticles; by encapsulating FPS-ZM1, this system effectively suppresses the RAGE pathway and steers macrophages toward the M2 phenotype, which is instrumental in accelerating angiogenesis and resolving the stalled inflammatory phase of diabetic wound healing (Sun et al., 2022). This strategy of targeting local hypoxia and oxidative stress is further refined through the use of ferrite and cerium-based oxides. Jonghoon Kim et al. developed MFC-MSNs (manganese ferrite and cerium oxide nanoparticles) to specifically address the hypoxic and ROS-rich synovial environment of rheumatoid arthritis (RA), inducing an M1-to-M2 transition that significantly alleviates joint inflammation (Kim et al., 2019; Ni et al., 2019b). The clinical transmissibility of such cerium/manganese oxide nanoparticles has been further improved by Tian Xia et al., who integrated these particles into microneedle arrays to deliver methotrexate (MTX), thereby ensuring targeted regulation of the macrophage balance within RA tissues (Ni et al., 2019b). The therapeutic potential of cerium-based nanoparticles, frequently referred to as nanoceria, is rooted in the reversible transition between Ce^3+^ and Ce^4+^ oxidation states on the particle surface. This valence fluctuation, coupled with the formation of oxygen vacancies, allows nanoceria to function as inorganic multi-enzyme mimetics. Specifically, nanoceria with a high Ce^3+^/Ce^4+^ ratio exhibit robust superoxide dismutase (SOD)-like activity for scavenging superoxide radicals, while those dominated by Ce^4+^ display enhanced catalase-like activity for decomposing hydrogen peroxide. In the chronic inflammation of the RA synovial microenvironment, nanoceria effectively break the inflammatory cycle by acting as high-capacity redox buffers that neutralize both mitochondrial and extracellular ROS species. This scavenging action results in the downregulation of redox-sensitive signaling cascades, particularly the NF-κ and MAPK pathways, which are critical for M1 polarization. By restoring synovial redox homeostasis, nanoceria do not merely provide antioxidant protection but actively create a permissive environment for the reprogramming of macrophages toward a reparative M2 phenotype, ultimately promoting tissue remodeling and long-term remission. The integration of photothermal therapy (PTT) with biochemical modulation represents another critical evolution in the use of alternative metal nanostructures. In the treatment of atherosclerosis, the multifunctional GSNPD system has been developed to target and eliminate pro-inflammatory macrophages via PTT, while its intrinsic anti-inflammatory properties inhibit any secondary inflammation typically induced by thermal stress (Lu et al., 2025). Similarly, the unique optical properties of noble metals like ruthenium are being exploited for synergistic immunomodulation. Xu Chen et al. designed the QRu-PLGA-RES-DS NPs core-shell complex, which utilizes quadrilateral ruthenium nanoparticles (QRuNPs) as a photothermal core; by orchestrating the controlled release of resveratrol (RES), this system reverses M1 polarization and enhances anti-inflammatory outcomes through photothermal synergy, offering a sophisticated nano-solution for RA management (Chen et al., 2019). Collectively, these studies underscore that by diversifying the metallic compositions of nanoparticles, researchers can tailor the redox-active and photophysical properties of the delivery system to precisely correct the macrophage-mediated imbalances inherent in chronic immune-related disorders.

The clinical utility of the MNPs discussed above is fundamentally determined by the balance between ROS-mediated signaling and pathological oxidative damage, a relationship best described as a concentration-dependent biological rheostat. Within a signaling window characterized by low to moderate ROS levels, redox-active platforms such as MnO_2_, Fe_3_O_4_, and MoS_2_ harness transient species as secondary messengers to activate redox-sensitive transcription factors, including NF-κB and Nrf2. This targeted activation facilitates controlled phenotypic shifts while preserving cellular viability. In contrast, excessive ROS accumulation initiates a transition toward indiscriminate damage, NLRP3 inflammasome activation, and eventual cell death. A defining characteristic of these metallic nanozymes is their capacity to act as redox buffers, which catalytically maintain the ROS pool within an optimal physiological window to ensure stable macrophage reprogramming without inducing off-target inflammatory injury (Table 9).

**Table 9:** Other metallic NPs act on macrophages to treat various diseases.

Nanoparticle	Effect on Macrophages	Key Mechanism	Disease Application	References
FZ@ZIF-67 (Co-MOF)	Promotes M2 polarization (anti-inflammatory)	Release of Co^2+^ promotes angiogenesis, FPS-ZM1 inhibits RAGE pathway, synergistically induces M2 phenotype	Diabetic wound healing	(Sun et al., 2022)
MFC-MSNs (MnFe₂O₄/CeO₂)	M1 → M2 repolarization (alleviating inflammation)	MnFe₂O₄ alleviates hypoxia, CeO₂ scavenges ROS, synergistic delivery of methotrexate (MTX)	Rheumatoid arthritis (RA)	(Kim et al., 2019)
BSA@NPs-MTX (Ce/MnOₓ)	M1 → M2 repolarization (regulating M1/M2 balance)	Ce/MnOₓ scavenges ROS, MTX for anti-inflammatory effects, transdermal delivery via microneedles (MN)	Rheumatoid arthritis (RA)	(Ni et al., 2019b)
GSNPD (Au NR@SiO₂/RSNO/DS)	Ablation of M1 macrophages, inhibits inflammation	Photothermal therapy (PTT) targets M1 macrophage ablation, NO release inhibits PTT side effects	Atherosclerosis	(Lu et al., 2025)
QRu-PLGA-RES-DS (Ruthenium nano)	M1 → M2 repolarization (anti-inflammatory)	Ruthenium nano core for photothermal synergy, resveratrol (RES) controlled release to modulate macrophage phenotype	Rheumatoid arthritis (RA)	(Chen et al., 2019)

### 3.10 Comparative Analysis and Strategic selection of MNP Platforms

While individual metallic nanostructures have specific roles, choosing the right platform requires a comparison of their strategic advantages as summarized in Table 10. This selection is fundamentally dictated by the disease microenvironment and the desired immune shift. For instance, Fe_3_O_4_ nanoparticles provide magnetic targeting for M1 polarization in oncology, whereas MnO_2_ particles act as catalytic engines to alleviate hypoxia or oxidative stress. In parallel, gold and silver nanoparticles serve as versatile scaffolds for tissue repair by inducing M2 reparative phenotypes. More specialized materials like MoS_2_ utilize enzyme like activity for chronic infections, while CaCO_3_ particles use pH responsive dissolution to target acidic tumor sites. These distinct mechanisms are the foundation for designing next generation immunotherapeutics.

**Table 10:** Comparative Summary of Metal-Based Nanoparticle Platforms.

Metal Platform	Primary Strengths	Polarization Preference	Limitations & Translational Hurdles	Safety & Toxicity Concerns
Gold (Au)	High stability; Precision LSPR control	M1 or M2 (Versatile)	High cost; Low biodegradability	Non-biodegradable; Long-term accumulation in organs
Silver (Ag)	Potent antimicrobial synergy	M1 → M2 (Reparative)	Concentration-dependent toxicity	Oxidative stress-induced DNA damage
Iron (Fe₃O₄)	MRI-guidance; Established clinical use	M2 → M1 (Immunogenic)	Iron overload risk	Alteration of iron homeostasis (Siderosis)
Manganese (MnO₂)	Catalytic oxygenation; Bio-responsive	M2 → M1 (Antitumor)	Potential for ionic leakage	Risk of Mn-induced neurotoxicity
Molybdenum (MoS₂)	Nanozyme activity (peroxidase-like); Tunable surface chemistry; Photothermal properties	M1 ↔ M2 (Switchable)	Industrial origin; Limited clinical translation data	Limited long-term safety data; Potential oxidative stress
Titanium (TiO₂)	Surface-topography control	M1 or M2 (Context-dependent)	Potential for chronic inflammation	TLR4-dependent systemic activation
Zinc (ZnO)	Chemosensitization; High loading	M2 → M1 (Immunogenic)	Rapid dissolution in acidic TME	Biphasic effects leading to ferroptosis
Calcium (CaCO₃)	High biocompatibility; pH-responsive	M2 → M1 (Antacid)	Poor intrinsic imaging contrast	Minimal (Systemic Ca homeostasis)

The translational potential of these platforms depends on balancing such therapeutic efficacy with rigorous safety profiles. Each metal species presents unique toxicological risks that must be managed. Gold is stable but non biodegradable, posing concerns regarding long term accumulation in the body. Silver provides potent antibacterial polarization but can cause off target ROS damage to healthy tissues. For transition metals like iron and manganese, the primary hurdle is maintaining metal ion homeostasis to avoid siderosis or neurotoxicity. Similarly, TiO_2_ and ZnO nanoparticles show concentration dependent responses where high doses may trigger unwanted proinflammatory cascades. Ultimately, strategic selection must evaluate biological fate and safety margins alongside polarization efficiency to ensure clinical viability.

## 4 Summary and future perspectives

MNPs have emerged as a primary focus for modulating macrophage polarization across diverse disease landscapes. However, the cross-study comparison of research findings remains challenging due to the lack of uniformity in experimental designs. The biological identity and immunomodulatory efficacy of MNPs are fundamentally dictated by the dynamic interplay between their physical dimensions, morphology, surface chemistry, and the spontaneous formation of a protein corona in biological media. To address these issues and facilitate interpretability, Table S5 in the Supporting Information provides a structured comparison of experimental parameters, including dose metrics, morphology, and surface chemistry, for representative systems. Upon exposure to physiological fluids, MNPs adsorb a complex layer of host proteins, creating a biological interface that masks the underlying synthetic properties and determines the nature of immune cell interactions. The composition and density of this corona, largely governed by the particle's initial size and surface charge, significantly modulate macrophage uptake kinetics and the subsequent direction of polarization. For instance, the selective recruitment of opsonizing proteins can amplify M1-like pro-inflammatory responses, whereas the adsorption of specific dysopsonins may facilitate immune evasion or steer macrophages toward reparative M2 phenotypes. Furthermore, particle geometry, exemplified by high-aspect-ratio structures like nanostars or nanorods, provides mechanical cues that physically deform the macrophage membrane to favor M2-like reparative phenotypes via elongation-induced mechanotransduction. Variability in macrophage sources and disease models further adds to the data complexity. To address these issues and facilitate interpretability, Table S5 in the Supporting Information provides a structured comparison of experimental parameters, including dose metrics, morphology, and surface chemistry, for representative systems.

This review systematically detailed the mechanisms by which MNPs influence macrophage destiny, primarily focusing on two distinct approaches: passive modulation and active targeting. Passive strategies capitalize on the MNPs' ability to scavenge reactive oxygen species (ROS) or directly interfere with intracellular signaling pathways (e.g., NF-κB, JAK/STAT) to suppress pro-inflammatory M1 polarization or promote anti-inflammatory M2 conversion. Active targeting, conversely, utilizes surface modification with specific ligands (e.g., peptides, antibodies) or disease-specific microenvironment triggers (e.g., pH, redox state) to deliver therapeutic cargos (drugs, genes) directly into macrophages, enabling highly efficient and selective phenotypic switching. The progress highlighted here demonstrates the profound potential of MNPs in enhancing immunotherapy, resolving chronic inflammation, and accelerating tissue repair.

Despite these exciting developments, the transition of MNP-based macrophage reprogramming from bench to bedside still faces several critical hurdles. The immediate focus must be placed on foundational safety and biological control. Crucially, as the strength of mechanistic evidence varies across the literature, we have introduced an EGS in Table S4 to distinguish between purely correlative findings and those validated through causal interventions. A deeper, unbiased understanding of the long-term biological fate and toxicological profile of MNPs is paramount. Future studies must employ advanced omics technologies, including single-cell RNA sequencing and spatial transcriptomics, to precisely map the dynamic molecular interplay between MNPs and macrophage subsets in vivo. Emphasis should be placed on investigating potential off-target effects and chronic toxicity, including clearance mechanisms and the induction of unwanted immune responses.

The second crucial area concerns therapeutic synergy and clinical translation. The future lies in integrating macrophage reprogramming with other leading therapeutic modalities. For cancer, this involves combining MNP-mediated M1 activation with checkpoint inhibitors, radiation therapy, or adoptive T-cell transfer to create a highly synergistic anti-tumor immune microenvironment. For non-cancer diseases, such as atherosclerosis or fibrosis, integrating MNPs with established small-molecule drugs to enhance their therapeutic index via macrophage-mediated drug delivery will be a key focus. However, bridging the translational gap requires moving beyond preclinical success to address the industrial and regulatory realities of drug development. A primary bottleneck is the large-scale, cGMP-compliant manufacturing of MNPs; maintaining batch-to-batch consistency in size, surface functionalization, and protein corona characteristics is technically demanding but essential for clinical-grade materials.

Furthermore, the pharmacokinetics and biodistribution of these systems are fundamentally shaped by their natural sequestration in the mononuclear phagocyte system (MPS), primarily the liver and spleen. While this provides a natural avenue for treating hepatic or splenic disorders, it complicates the targeting of distal tissues and necessitates a sophisticated understanding of clearance half-lives to avoid long-term toxicity and metal ion dyshomeostasis. Clinically relevant benchmarks, such as the use of superparamagnetic iron oxide (SPIO) nanoparticles for both imaging and modulating tumor-associated macrophages, represent the current vanguard of this field, yet many other metal-based platforms remain in early investigative stages. Finally, regulatory considerations dictate that we move toward standardized in vivo characterization techniques to satisfy the rigorous safety requirements for human trial approvals.

Beyond experimental standardization, the clinical viability of MNPs depends on resolving the tension between targeting specificity and off-target immunomodulation. Since these materials naturally sequester in clearance organs, researchers must evaluate whether local reprogramming at the disease site triggers unintended systemic immune shifts. To address this, we recommend the Minimal In Vivo Assessment Set in Table S6 to standardize the evaluation of organ-specific accumulation, cytokine levels, and immune cell profiling. Adopting such a standardized safety panel is necessary to distinguish truly targeted nanotherapeutics from those that might cause broad disruptions to systemic immune homeostasis. In conclusion, MNPs are poised to transform macrophage-centric therapeutics. By tackling the challenges related to manufacturing scalability, pharmacokinetics, and clinical safety, the next generation of metal-based nanomedicines will unlock the full potential of macrophage reprogramming for managing currently intractable diseases.

## Funding and acknowledgments

This study was supported by the 10.13039/501100001809National Natural Science Foundation of China (No. 82204319), National Health and Medical Research Council (NHMRC) Early Career Fellowship (Grant No. 1112258) and Western Sydney University Vice-Chancellor's Senior Research Fellowship.

## CRediT authorship contribution statement

**Xing Du:** Writing – original draft, Visualization, Formal analysis. **Kai Ding:** Writing – original draft, Investigation. **Zehao Mao:** Writing – original draft, Investigation. **Jiangyu Li:** Writing – original draft, Investigation. **Yongmei Zhao:** Writing – review & editing, Supervision, Conceptualization. **Tianqing Liu:** Writing – review & editing, Conceptualization.

## Declaration of competing interest

The authors declare the following financial interests/personal relationships which may be considered as potential competing interests:

## Data Availability

No data was used for the research described in the article.

## Appendix A Supplementary data

Supplementary material: 1. Table S1: Exact Search Strings Used in Bibliographic Databases (PubMed, Web of Science, and Scopus) for studies published between January 2015 and April 2026. 2. Table S2: Categorized Summary of Included References by Nanoparticle Type. 3. Table S3: Map of Biological Readouts to Phenotype Interpretation, detailing surface markers, enzymes, cytokines, and transcription factors for M1-like and M2-like macrophages. 4. Table S4: Grading of Key Mechanistic Pathways in MNP-Mediated Polarization based on causal validation and evidence levels. 5. Table S5: Standardized Comparison of Representative MNP-Mediated Polarization Studies. 6. Table S6: Recommended Minimal In Vivo Assessment Set for MNP Translation, detailing proposed methodology and standard readouts for organ accumulation and immune profiling. 7. Table S7: Analysis of Core-Driven vs. Ligand-Driven Polarization in Gold Nanoparticle (AuNP) Systems across mechanotransduction, photothermal, and delivery categories.
