# Supplementary material for: Metal-based nanoparticles for reprogramming macrophage polarization: Advances in immunomodulatory nanotherapeutics
Source: Int J Pharm X. 2026 May 7;11:100564. doi: 10.1016/j.ijpx.2026.100564 (PMC13200098; doi:10.1016/j.ijpx.2026.100564)
Supplement: Supplementary file 1 — Supplementary material: 1. Table S1: Exact Search Strings Used in Bibliographic Databases (PubMed, Web of Science, and Scopus) for studies published between January 2015 and April 2026. 2. Table S2: Categorized Summary of Included References by Nanoparticle Type. 3. Table S3: Map of Biological Readouts to Phenotype Interpretation, detailing surface markers, enzymes, cytokines, and transcription factors for M1-like and M2-like macrophages. 4. Table S4: Grading of Key Mechanistic Pathways in MNP-Mediated Polarization based on causal validation and evidence levels. 5. Table S5: Standardized Comparison of Representative MNP-Mediated Polarization Studies. 6. Table S6: Recommended Minimal In Vivo Assessment Set for MNP Translation, detailing proposed methodology and standard readouts for organ accumulation and immune profiling. 7. Table S7: Analysis of Core-Driven vs. Ligand-Driven Polarization in Gold Nanoparticle (AuNP) Systems across mechanotransduction, photothermal, and delivery categories. [file mmc1.docx]

**Metal-Based Nanoparticles for Reprogramming Macrophage Polarization: Advances in Immunomodulatory Nanotherapeutics**

Xing Du^1^, Kai Ding^1^, Zehao Mao^1^_,_ Jiangyu Li^1^, Yongmei Zhao^1*^, Tianqing Liu^2*^

^1^ School of Pharmacy, Nantong University, Nantong, China

^2^ School of Science, Western Sydney University, Penrith, NSW 2751, Australia

**Corresponding Authors**

*** Email: ymzhao@ntu.edu.cn**

[**michelle.tianqing.liu@gmail.com**](mailto:michelle.tianqing.liu@gmail.com)

To capture the functional evolution of metallic nanoparticles (MNPs) in immunotherapy, we searched PubMed, Web of Science, and Scopus for relevant studies published between January 2015 and April 2026. This period was selected to encompass the technological transition from simple carriers to active immunomodulatory platforms. Using a combination of metal specific keywords and functional descriptors (e.g., "reprogramming" or "phenotypic switching"), we identified papers exploring the interactions between various metals (Au, Ag, Fe, etc.) and macrophage behavior. Our selection prioritized studies offering clear mechanistic data including signaling pathway interference or ROS modulation, and those demonstrating efficacy in clinical models. Conversely, papers focusing on general cytotoxicity without a biological framework, non-metallic organic systems, or non-English literature were excluded from this review.

**Table S1 Exact Search Strings Used in Bibliographic Databases**

| **Category** | **Primary Search String** |
| --- | --- |
| General MNPs | ("Metal-based nanoparticles" OR "MNPs" OR "Inorganic nanoparticles") AND ("macrophage polarization" OR "immunomodulation" OR "M1/M2") |
| Gold (Au) | ("Gold nanoparticles" OR "AuNPs" OR "Au nanostars") AND ("macrophage reprogramming" OR "phenotypic shift" OR "M1/M2 balance" OR "tumor-associated macrophages" OR "tissue repair") |
| Silver (Ag) | ("Silver nanoparticles" OR "AgNPs") AND ("M1 to M2 polarization" OR "anti-inflammatory" OR "wound healing" OR "antibacterial" OR "ROS scavenging") |
| Iron Oxide (Fe) | ("Iron oxide" OR "Fe_3_O_4_" OR "SPIO" OR "superparamagnetic iron oxide") AND ("TAMs" OR "tumor-associated macrophages" OR "MRI tracking" OR "magnetic hyperthermia" OR "M2-to-M1 repolarization") |
| Mo & Mn | ("MoS_2_" OR "Molybdenum disulfide" OR "MnO_2_" OR "Manganese dioxide") AND ("nanozyme" OR "ROS scavenging" OR "photothermal therapy" OR "hypoxia relief" OR "STING pathway") |
| Zn & Ti | ("ZnO" OR "Zinc oxide" OR "TiO_2_" OR "Titanium dioxide") AND ("osteogenesis" OR "tumor microenvironment" OR "photocatalysis" OR "bone regeneration" OR "implant surface") |
| Calcium (Ca) | ("CaCO_3_" OR "Calcium carbonate") AND ("pH-responsive" OR "acidity neutralization" OR "tumor microenvironment" OR "oxygen generation" OR "bone repair") |
| Other Metals | ("Cobalt" OR "Cerium oxide" OR "Ruthenium" OR "Cu_2_O" OR "ZIF-67") AND ("metal-organic framework" OR "nanozyme" OR "photothermal therapy" OR "M1/M2 polarization") |

**Table S2. Categorized Summary of Included References**

| **Nanoparticle Type** | **Total References** | **Key Reference Range** |
| --- | --- | --- |
| Gold (Au) | 38 | [84–118, 125–127] |
| Silver (Ag) | 22 | [119–140] |
| Molybdenum (MoS_2_) | 11 | [141–151] |
| Titanium (TiO_2_) | 8 | [152–159] |
| Zinc (Zn-based) | 4 | [160–163] |
| Manganese (MnO_2_) | 15 | [164–178] |
| Iron Oxide (Fe_3_O_4_) | 17 | [179–195] |
| Calcium (CaCO_3_) | 6 | [196–201] |
| Other Metals (Co, Ce, Ru) | 5 | [202–206] |

**Table S3. Map of Biological Readouts to Phenotype Interpretation**

| **Category** | **Readout/Marker** | **Associated Phenotype** | **Interpretation/Function** | **Pitfalls & Context Dependence** |
| --- | --- | --- | --- | --- |
| Surface Markers | CD80, CD86, CD40 | M1-like | Co-stimulation for T-cell activation | Marker Overlap: CD86 is also a characteristic marker for the M2b subtype. |
|  | CD206, CD163 | M2-like | Scavenging, tissue remodeling, and repair | Expression Variance: CD206 expression can fluctuate based on the specific IL-4/IL-13 stimulus ratio. |
| Enzymes | iNOS | M1-like | Generation of NO for microbicidal/tumoricidal activity | Context: In certain TME settings, M1-like cells may paradoxically produce pro-tumor factors like IL-1β. |
|  | Arg-1 | M2-like | Collagen synthesis and suppression of T-cell proliferation | Species Difference: Arg-1 is a robust marker in murine models but is less definitive in human macrophages. |
| Cytokines | IL-12, TNF-α, IL-23 | M1-like | Promoting Th1 responses and anti-tumor immunity | Systemic Risk: Excessive M1 induction can lead to secondary bacterial susceptibility. |
|  | IL-10, TGF-β | M2-like | Immunosuppression and resolution of inflammation | Pro-fibrotic Risk: Chronic M2 polarization is a primary driver of pulmonary and organ fibrosis. |
| Transcription | STAT1, IRF5, NF-κB | M1-like | Pro-inflammatory signaling cascade | Pathway Crosstalk: NF-κB activation is common to multiple inflammatory stimuli. |
|  | STAT3, STAT6, IRF4 | M2-like | Anti-inflammatory and reparative signaling | Context Dependence: STAT3 signaling is highly active in TAMs within the TME. |

**Table S4. Grading of Key Mechanistic Pathways in MNP-Mediated Polarization**

| **Nanoparticle Feature** | **Proposed Mechanism** | **Causal Validation (Level 1)** | **Evidence Grade** |
| --- | --- | --- | --- |
| Magnetic (Fe₃O₄) | JAK/STAT3 signaling blockade | Yes: siRNA-mediated STAT3 silencing | High |
| Mechanical (AuNPs) | RGD-caging/Adhesion cues | Yes: Reversible magnetic manipulation of ligands | High |
| Catalytic (MnO₂) | Hypoxia relief via O₂ supply | Partial: Blockade of HIF-1α downstream pathways | Moderate |
| Surface (Au/Ag) | TLR4/NF-κB inhibition | Yes: Use of specific inhibitors like punicalagin or p65 citrullination | High/Mod |
| Epigenetic (AuNPs) | m6A/ATG5 pathway regulation | Partial: m6A methylation level monitoring | Moderate |
| Metabolic (MoS₂) | ROS scavenging/Mito-protection | Partial: Targeted delivery to mitochondria via TPP | Moderate |

**Table S5. Standardized Comparison of Representative MNP-Mediated Polarization Studies**

| **Nanoparticle Type** | **Particle Size / Surface Charge** | **Surface Coating** | **Dose Metric & Duration** | **Macrophage Source** | **Disease Model / Route** | **Endotoxin Testing** | **Ref.** |
| --- | --- | --- | --- | --- | --- | --- | --- |
| AuNPs (PEG-AuNPs) | 20 nm / Neutral | PEG | 0.02–0.1 mg/kg; 21 days | TAMs (Hepa1-6) | Liver Cancer / Subcutaneous | Reported | [102] |
| AuNPs (P12-AuNPs) | ~30 nm / Negative | P12 Peptide | 10 µg/mL; 24 h | RAW 264.7 | Acute Lung Injury / Intranasal | Not Specified | [86] |
| AgNPs (FA-AgNPs) | ~50 nm / Negative | Folic Acid | 5 mg/kg; 14 days | RAW264.7 | Rheumatoid Arthritis / Intra-articular | LAL Assay | [122] |
| MnO₂ (HMMDN) | ~150 nm / Negative | PM (Membrane) | 5 mg/kg; 15 days | M2-TAMs | Tumor Immunotherapy / I.V. | Reported | [164] |
| Fe₃O₄ (FPA) | ~80 nm / Positive | PDA-Ag | 200 µg/mL; 24 h | RAW 264.7 | MRSA Infection / Subcutaneous | Not Specified | [191] |
| CaCO₃ (CAT\@CaCO₃) | ~200 nm / Negative | Catalase | 10 mg/kg; 25 days | M2-TAMs | Broad Antitumor / I.V. | Reported | [198] |
| MoS₂ (NS/Sericin) | Nanosheet / Neutral | Sericin | 100 µg/mL; 24 h | RAW 264.7 | Infected Wound / Topical | Not Specified | [150] |

**Table S6. Recommended Minimal In Vivo Assessment Set for MNP Translation**

| **Assessment Category** | **Proposed Methodology** | **Standard Readouts** | **Rationale for Inclusion** |
| --- | --- | --- | --- |
| Organ Accumulation | ICP-MS/OES & Whole-body Imaging | % ID/g in Liver, Spleen, Kidney, and Heart | To quantify passive sequestration and verify targeting efficiency. |
| Systemic Cytokines | ELISA or Multiplex Immunoassays | Serum TNF-α, IL-6, IL-12, and IL-10 | To monitor for systemic inflammatory responses or broad immunosuppression. |
| Tissue Histology | H\&E & Masson's Trichrome | Morphological damage, necrosis, or fibrosis in liver/spleen | To assess direct physical toxicity and long-term tissue remodeling. |
| Immune Profiling | Flow Cytometry (FACS) or CyTOF | M1/M2 ratios and T-cell status in non-target organs | To detect off-target polarization shifts that may compromise systemic immunity. |

**S7 Analysis of Core-Driven vs. Ligand-Driven Polarization in AuNP Systems**

| **Study Category** | **Core Property Utilized** | **Primary Mechanism** | **Dominant Driver** | **Ref.** |
| --- | --- | --- | --- | --- |
| Mechanotransduction | Shape/Anisotropy (Stars/Rods) | Physical membrane deformation & cytoskeleton remodeling | Material Core | [87, 94] |
| Photothermal | LSPR (NIR-responsiveness) | Thermal-induced cytokine release & M1-switching | Material Core | [98, 105] |
| Multivalent Presentation | High Surface Area / Thiol Chemistry | Clustered receptor signaling (e.g., TLR4/CD86) | Hybrid | [85, 113] |
| Cargo Delivery | Biocompatibility / Stability | Controlled release of siRNA, miRNA, or drugs | Cargo/Ligand | [90, 111] |
| Imaging & Tracking | High Electron Density / CT Contrast | Real-time monitoring of TAM infiltration | Material Core | [102] |
